# Assessment of Stability of Preservation Techniques for Strawberry Soil Microbiomes: Exploring the Biotechnological Relevance for Sustainable Agriculture

**DOI:** 10.3390/microorganisms14071547

**Published:** 2026-07-15

**Authors:** Federico Sbarra, Marco Garello, Andrea Visca, Filippo Sevi, Francesco Aloi, Silvia Tabacchioni, Manuela Costanzo, Luciana Di Gregorio, Eleonora Colantoni, Lorenzo Nolfi, Samuel Mondy, Luca Simone Cocolin, Davide Spadaro, Giovanna Cristina Varese, Annamaria Bevivino

**Affiliations:** 1 Department for Sustainability, ENEA, Italian National Agency for New Technologies, Energy and Sustainable Economic Development, Casaccia Research Center, Via Anguillarese 301, 00123 Rome, Italy; federico.sbarra@unito.it (F.S.); andrea.visca@enea.it (A.V.); filippo.sevi@enea.it (F.S.); silvia.tabacchioni@enea.it (S.T.); manuela.costanzo@enea.it (M.C.); luciana.digregorio@enea.it (L.D.G.); eleonora.colantoni@enea.it (E.C.); lorenzo.nolfi@unitus.it (L.N.); 2 Department of Life Sciences and Systems Biology (DBIOS), University of Turin, Viale Mattioli, 25, 10125 Turin, Italy; cristina.varese@unito.it; 3 Department of Agricultural, Forest and Food Sciences (DISAFA), University of Turin, Largo Paolo Braccini 2, 10095 Grugliasco, Italy; marco.garello@unito.it (M.G.); francesco.aloi@unito.it (F.A.); lucasimone.cocolin@unito.it (L.S.C.); davide.spadaro@unito.it (D.S.); 4 Department of Agriculture and Forest Sciences, University of Tuscia, 01100 Viterbo, Italy; 5 Institut Agro Dijon, Agroécologie, INRAE, Université Bourgogne-Franche-Comté, 21000 Dijon, France; samuel.mondy@inrae.fr

**Keywords:** soil microbiome, sample preservation, metabolic fingerprinting, microbial community stability, microbiome biobanking

## Abstract

Preserving ex situ soil microbial communities is essential for bacterial biodiversity conservation in microbiome biobanking, accurate microbiome research, and future biotechnological applications in sustainable agriculture. The effect of three soil preservation methods—cryopreservation at −80 °C, refrigeration at 4 °C, and lyophilization—on the viability, diversity and metabolic functionality of the soil microbiome was evaluated using soil from untreated and solarized strawberry fields stored at 0, 6 and 12 months. Microbial viability, ecological strategies, functionality, and diversity were assessed using plate counts, r/K strategist profiling, BIOLOG EcoPlates, and 16S rRNA/ITS gene amplicon-based sequencing, respectively. Cryopreservation outperformed the other preservation strategies by generally maintaining total bacterial counts, especially in control soil, preserving the balance between oligotrophic and copiotrophic communities, and sustaining high alpha diversity indices over 12 months. Refrigeration provided moderate preservation, though microbial viability and community stability declined over time. In contrast, lyophilization significantly reduced microbial abundance, altered community composition, and diminished metabolic diversity and substrate utilization. Beta diversity analyses confirmed that cryopreserved samples remained similar in microbial structure to the initial timepoint, while lyophilized samples diverged substantially. Functional analyses also revealed reduced metabolic activity in lyophilized soils, underscoring the negative impact on the microbiome’s ecological roles. Overall, the results of this study identify cryopreservation as the most effective storage method for maintaining soil microbiome integrity, providing critical guidance for preserving soil samples in microbiome research and sustainable agricultural biotechnology.

## 1. Introduction

Soil microbial communities play fundamental roles in nutrient cycling, plant health, and soil structure maintenance, directly influencing crop productivity and resilience to biotic and abiotic stresses [1]. The stability and preservation of soil microbiomes are essential for advancing sustainable agriculture, particularly for economically important crops like strawberries [2]. To maximize the long-term benefits from preserved soil samples, as mixed microbiomes, it is crucial to maintain their viability, genetic stability, and functional integrity during preservation [3]. Inadequate preservation can lead to shifts in community composition, loss of metabolically active taxa, and alterations in key functional traits, potentially biasing downstream microbiological, molecular, and ecological analyses [4,5]. With growing interest in microbiome-based strategies to enhance soil fertility, disease resistance, and plant performance, accurate characterization and long-term conservation of complex microbial communities are essential to ensure that preserved samples remain representative of in situ soil conditions [6]. Long-term storage of mixed cultures and whole microbiomes is crucial for maintaining the authenticity of the sample in microbiome biobanking and for the development and application of microbiome-based innovations [7]. However, the identification of proper, effective storage methods that retain native microbial diversity and functional potential is crucial for advancing microbial ecology and biotechnology [8]. From both a biotechnology and bioeconomy perspective, effective ex situ and in situ preservation of microbial biodiversity is a critical step toward harnessing the beneficial potential of microorganisms and supporting sustainable agricultural applications.

Storage conditions can selectively impact microbial taxa composition [9]. Inconsistent storage practices can introduce biases in microbial community analyses, hinder cross-study comparisons, and compromise the development of reproducible microbial formulations for field applications. While numerous protocols and techniques exist for preserving pure cultures, relatively little attention has been given to preserving intact samples and their associated microbiomes [3]. Refrigeration at 4 °C is one of the most used approaches for short-term soil microbiome preservation due to its practicality and ability to moderately slow microbial metabolic activity. Studies have shown that short-term storage, typically up to 2 to 4 weeks, under refrigerated conditions generally maintains microbial community structure and DNA integrity with minimal disruption, making it suitable for transport and processing delays in field-based studies [10,11]. However, as storage duration extends, even at low temperatures, microbial communities can undergo compositional shifts, reduced viability, and changes in functional parameters such as respiration and enzyme activity [12]. In contrast, lyophilization, widely adopted for pure cultures and environmental samples, offers clear advantages, particularly in terms of shelf life, ease of handling, and reduced risk of contamination, providing stable preservation of nucleic acids and facilitating transport at room temperature [4,13], similar to the archived or air-dried soil [14,15,16]. However, the freeze-drying process can induce osmotic and oxidative stress, thus damaging cellular membranes and leading to reduced viability, especially among sensitive taxa. To mitigate these effects, protective agents (known as lyoprotectants, including trehalose, skim milk, or glycerol) are often added before drying to stabilize cellular components during dehydration and rehydration [17]. Nevertheless, the effectiveness of these additives is often species-specific, and their overall impact on the viability, composition, and functional potential of complex microbial consortia remains uncertain, posing challenges for reproducibility in microbiome-based applications [18,19,20]. Cryopreservation, typically performed at ultra-low temperatures such as −80 °C or in liquid nitrogen (−196 °C), is another widely used strategy for long-term preservation of microbial communities [4,21]. It effectively halts metabolic activity and stabilizes cellular structures, thereby maintaining microbial viability and genetic integrity over extended periods. When combined with cryoprotectants such as glycerol or dimethyl sulfoxide (DMSO), this method minimizes ice crystal formation and osmotic damage during freeze–thaw cycles [18,22]. Although cryopreservation is highly effective for isolated strains or synthetic microbial consortia, its application to complex soil microbiomes can be more challenging due to heterogeneous microbial responses [11]. Certain taxa may be more susceptible to freezing stress, leading to community shifts upon thawing and potentially compromising the functional stability of the preserved microbiome.

Strawberries are highly sensitive to soil quality and environmental stressors [23], both of which are influenced by the composition and diversity of the soil microbiome [24]. Strawberry cultivation stands to benefit from microbiome-informed management strategies due to its high susceptibility to soilborne pathogens and its strong dependence on soil health. The microbial imbalance, called dysbiosis, observed in unhealthy plants highlights the importance of maintaining a stable microbial community to enhance crop productivity [25,26]. In this context, the expanding use of microbial inoculants, disease-suppressive approaches, and soil health diagnostics highlights the need for reliable methods to preserve soil samples for both fundamental research and applied microbiome studies. Here, we systematically evaluate the long-term effects of three widely used preservation methods of soil samples collected from solarized and untreated strawberry fields—refrigeration at 4 °C, cryopreservation at −80 °C and lyophilization (freeze-drying)—on key microbial parameters, including viability, ecological strategy distribution, diversity, and functional potential assessed using plate counts, r/k strategist profiling, 16S rRNA/ITS gene amplicon-based sequencing and BIOLOG EcoPlates system, respectively. Soil samples were collected from solarized and untreated strawberry fields and analyzed immediately after sampling and after 6 and 12 months of storage. Our results demonstrate that the choice of preservation method profoundly shapes microbial community profiles, with important implications for microbiome-based research and agricultural biotechnology. Selecting preservation strategies that minimize artificial shifts is therefore essential for accurate microbiome characterization in strawberry systems, where soil dynamics directly control plant health, disease suppression, and productivity. Overall, this work highlights how long-term preservation methods significantly influence the structural, functional, and ecological integrity of soil microbial communities, emphasizing the need for optimized storage strategies to ensure reliable microbiome characterization in strawberry agroecosystems.

## 2. Materials and Methods

### 2.1. Soil Sampling and Storage

Soil samples were collected from a strawberry (*Fragaria x ananassa*) tunnel near Boves (Cuneo, Italy; 44°20′51.1″ N 7°32′04.2″ E) from two distinct plots: one treated with an innovative solarization technique (Solin^®^ method) [27] and one untreated control plot. The solarization treatment consisted of spraying a biochar-based coating on the soil surface, followed by covering with a multilayer thermal film for a duration of 30 days during the summer season (July 2023) [27]. Sampling followed the standard operating procedures (SOPs) for soil microbiome studies [28]. Briefly, for each plot (treated and untreated), six plants were randomly selected. From each plant, bulk soil was collected according to the previously established SOPs. The six fresh soil samples were pooled to form a single composite sample, and from this soil, three 50 g aliquots for each timepoint and storage condition (21 total aliquots) were collected in 50 mL sterile Falcon tubes and processed in accordance with the selected storage protocol, ensuring sufficient material for both downstream analyses and the future setup of microbial consortia. Importantly, these aliquots were prepared as strictly independent storage aliquots at Time 0. At each designated timepoint (6 and 12 months), dedicated containers were retrieved and analyzed. No repeated sampling from the same container or freeze–thaw cycles occurred during the storage period, ensuring that statistical analyses of the technical replicates (i.e., independent storage aliquots) were not biased by sample handling. For refrigerated storage (4 °C), soil was kept in the dark inside a fridge; for cryopreservation (−80 °C), soil samples were immediately transferred to a −80 °C freezer; for lyophilized storage, soil samples were first frozen at −80 °C, then lyophilized using a ScanVac Coolsafe (Labogene, Lillerød, Denmark) and finally stored at room temperature in the dark.

### 2.2. Total Microbial Counts and Eco-Physiological Distribution

To assess the viability of the bacterial and fungal communities over the storage period, the number of colony-forming units (CFUs) was determined at the beginning of storage, after 6 months, and after 12 months. For fungi, at each timepoint and for each storage protocol, three technical replicates of 10 g of dried soil equivalents were transferred into a flask with 90 mL of sterile Phosphate-Buffered Saline (PBS) with 1 g of glass beads, and the resulting suspension was stirred for 1 h at 250 rpm. Three technical replicates (aliquots) were performed for each soil sample. Subsequently, the soil suspension was used to prepare 10-fold serial dilutions (in sterile 0.9% NaCl) up to 10^−4^, and for each dilution, 400 μL of soil suspension was plated on 15 cm plates containing either Dichloran Rose Bengal Chloramphenicol Agar (DRBCA) or Malt Extract Agar (MEA) supplemented with 250 mg/L ampicillin and 250 mg/L streptomycin. The suspension was spread uniformly over the plate surface using a sterile loop. Plates were then incubated in the dark at 28 °C for 7 days, and at the end of the incubation period, the number of colonies was counted and expressed as CFU per gram of soil.

For bacteria, at each timepoint and for each storage protocol, soil suspension was serially diluted with sterile saline solution (9 g L^−1^ NaCl) from 10^−1^ up to 10^−6^. The resulting dilutions obtained were plated on 0.1% Tryptic Soy Broth (TSB; Difco) amended with 15 g/L agar (0.1% TSA) supplemented with 100 µg/mL of cycloheximide (Sigma) to inhibit fungal growth. Plates were then incubated at 28 °C for 6 days. Colonies were counted daily for up to six days to capture both fast- and slow-growing bacteria. The number of colonies on plates with countable ranges (typically 30–300 colonies) was used to calculate the bacterial load, expressed as colony-forming units per gram of soil (CFU/g). Colonies were enumerated at 2 and 6 days of growth on 0.1 TSA. Bacterial colonies appearing at day 2 were classified as r-strategists, whereas the newly appearing colonies between day 2 and 6 were defined as K-strategists. For each storage condition and each timepoint, the distribution of r- and K-populations was given as a proportion (%) of total counts [29]. To evaluate the changes in the structure of the soil microbial community, the eco-physiological index (EPi) was used [30] and calculated using the equation:

(1) $$EPi=-\sum \left(Pi\;*\;{log}_{e}Pi\right)$$

where *Pi* represents the proportion of each bacterial class, namely r- and K-strategists, relative to the total CFU counts after 6 days of incubation, with EP_min_ = 0. Percentage data of EP index value were *logit*-transformed as described in Bevivino et al. [29]. Higher values of the EP index imply a more even distribution of proportions of bacteria developing on different days, i.e., different classes of bacteria. Microbial count data (CFU) were transformed to log10 (x + 1) to obtain the homogeneity of variance. Data were analyzed using one-way ANOVA with Tukey’s Honestly Significant Difference (HSD) test for multiple comparisons. The *p*-value threshold was set to 0.05 to identify statistically significant differences in the viable community fraction.

### 2.3. Community-Level Metabolic Profile

The functional metabolic potential of soil microbial communities was evaluated using the BIOLOG EcoPlate system (Catalog #1506–EcoPlate–BIOLOG Inc., Hayward, CA, USA). The assay was performed according to the recommendations of Cook et al. (2006) [31] with minor modifications. Briefly, five grams of dry weight equivalent of soil samples were preliminarily incubated in autoclaved 100-mL flasks containing 4.5 mL of sterile 1.8% Sodium Pyrophosphate (0.18% (*w*/*v*) Na_4_P_2_O_7_ × 10 H_2_O), 40.5 mL of 25% Ringer Solution, and 1 g of sterile glass beads (2-mm diameter) at 28 °C for 1 h under controlled agitation (180 r.p.m.). Two technical replicates (aliquots) were performed for each soil sample along all the preservation methods involved during the experiment. The soil suspension was then transferred to a sterile 50 mL plastic tube (avoiding the glass beads) and centrifuged at 500× *g* for 10 min. Soil supernatant (approx. 10^4^ CFUs mL^−1^) was used to inoculate the ECO-plates (Biolog Inc., Hayward, CA, USA) with 120 μL in each well. The ECO-plates with lids were wrapped in tin foil and incubated at 28 °C in the dark under humid conditions. Absorbance at 590 nm was read at 24-h intervals until the color development in each well reached the plateau [31]. The absorbance of each well was corrected by the subtraction of the optical density (OD590) of the well containing water. Average well color development (AWCD) was determined by calculating the mean of every well’s absorbance value at each reading time with the following formula:

(2) $$AWCD=\frac{\sum_{i=1}^{N}{OD}_{i}}{N}$$

where OD_i_ is the absorbance value of each carbon source containing well corrected with blank, while N is the number of substrates, which in this case is N = 31 [32].

Multivariate statistical analyses were performed to examine differences in functional metabolic profiles among preservation treatments and timepoints (0, 6, and 12 months). Principal Component Analysis (PCA) was carried out on the normalized absorbance matrix to identify the main axes of variance and to visualize clustering patterns of samples according to their carbon substrate utilization profiles. The PCA biplot was constructed using the ten most contributing substrates to the first two principal components to aid in the interpretation of functional differentiation. The variance explained by each axis was reported in the respective figure captions.

To further evaluate functional community dissimilarity among treatments, Principal Coordinate Analysis (PCoA) was conducted using Bray–Curtis dissimilarity distances calculated from the normalized BIOLOG data. The ordination plot was used to visualize clustering patterns according to preservation methods and storage duration. Statistical differences in community-level functional structure were assessed using the Kruskal–Wallis and Dunn’s post hoc tests, with Bonferroni correction applied for multiple comparisons. All analyses and visualizations were performed in R (version 4.5.1) [33] using the vegan (version 2.7.5) and ggplot2 (version 4.0.0) packages.

### 2.4. DNA Extraction and Sequencing

DNA extraction was performed using the commercial DNeasy PowerSoil Pro Kit (Qiagen, Hilder, Germany), according to the previously established and validated SOPs [28,34]. At each timepoint, for each storage condition and each soil, three independent soil aliquots were retrieved and processed, resulting in three technical replicates of the original pool, which were sequenced independently.

Sequencing was performed at the Nuova Genetica Italiana sequencing center (Villa Guardia, Como, Italy) on an Illumina NextSeq2000 using a 2 × 300 paired-end kit. For the fungal communities, the ITS3_KYO2 (5′-GATGAAGAACGYAGYRAA-3′) [35] and ITS4ngs (5′-TCCTCCGCTTATTGATATGC-3′) [36] primer pair was used to amplify the ITS2 region of the Internal Transcribed Spacer (ITS), while for the bacterial/archaeal communities the Pro341f (5′-CCTACGGGNBGCASCAG-3′) and Pro805r (5′-GACTACNVGGGTATCTAATCC-3′) [37] primer pair was used to amplify the V3-V4 region of the 16S rRNA gene.

Raw sequence data reported in this study were deposited in the European Nucleotide Archive (ENA) under project accession number PRJEB107956, also mirrored to the National Center for Biotechnology Information (NCBI) “Sequence Read Archive” (SRA).

### 2.5. Bioinformatics Analysis

Metabarcoding data were analyzed using the QIIME2 suite (version 2023.9), with separate analyses for fungi and bacteria. In short, raw reads were imported, and adapter sequences were removed using Cutadapt (version 4.5) with default parameters. Subsequently, trimmed sequences were processed with DADA2 (version 1.26) to recover the Amplicon Sequence Variants (ASVs) associated with each sample. Detailed read counts for each sample are reported in Supplementary Tables S1 and S2. For this step, quality filtering was performed using default parameters (e.g., maxEE = 2), while the forward and reverse truncation lengths were completely turned off (set to 0) to maximize read retention for both bacterial and fungal amplicons. To assign a taxonomic identity to these ASVs, a naive-Bayes classifier was trained on the UNITE database, v10.0 [38] for fungi, and another naive-Bayes classifier was trained on the Rescript-preprocessed SILVA database, v138 [39] for bacteria, respectively. Assignment was performed with a confidence cutoff of 0.9. Based on the results of taxonomic assignment, retrieved features were filtered to remove off-target organisms: for bacterial samples, contamination from mitochondrial and plastidial sequences was removed, while for fungal samples, all non-fungi sequences were removed.

Alpha diversity was evaluated based on the Shannon index, the number of observed features, the Pielou index of evenness, and the Simpson Index of diversity, while for beta diversity the Bray–Curtis dissimilarity was used. For the calculation of alpha and beta diversity, samples were normalized using the Scaling with Ranked Subsampling (SRS) [40] as implemented in the eponymous QIIME2 plugin (version 2021.4.0). For fungi, the normalization value was set at 49,000, while for bacteria, the normalization value was set at 42,000, using the same values for both soils. These specific SRS thresholds were selected to maximize the retention of sequence data while accommodating the different minimum sequencing depths yielded by the respective fungal and bacterial libraries, ensuring robust diversity estimations without dropping low-read samples. Calculation of the alpha and beta diversity metrics was performed with the standard q2-diversity module (version 2023.9). In addition, for beta diversity, dimensionality reduction was performed using the Principal Coordinates Analysis (PCoA) approach, as also implemented in the q2-diversity module.

To focus on the most representative taxa, the top 16 genera were selected based on overall mean abundance across all samples. For these, standardized z-scores were calculated to enable cross-genus comparison of abundance shifts. The z-score for each genus was computed as follows:

(3) $$z_{i}=\frac{x_{i}-x_{mean}}{s}$$

where *x_i_* is the mean abundance of genus *i* in a given sample or treatment, *x_mean_* is the mean abundance across all samples for that genus, and *s* is the corresponding standard deviation. This normalization expresses relative deviations in units of standard deviation, enabling visualization of enrichment (positive z) or depletion (negative z) events.

Differential abundance testing was conducted in R using ANCOM-BC2 (ancombc2, ANCOMBC package (version 2.12.1) on a taxon-by-sample count matrix with matched sample metadata. The explanatory variable *Class* (Time × Storage) was included in the fixed-effects model and specified as the grouping factor for treatment-level contrasts. For each taxon, the analysis returned estimated log-fold changes, standard errors, Wald statistics, and corresponding *p*-values and q-values, which were used to examine differential abundance patterns across conservation conditions, with emphasis on contrasts against *Time 0*. For the microbial diversity associated with alpha diversity, the presence of statistically significant differences was evaluated using a one-way ANOVA followed by Tukey’s Honestly Significant Differences (HSD) post hoc test, with a significance threshold of 0.05. For the microbial diversity associated with beta diversity, the presence and magnitude of statistically significant effects associated with the different variables were assessed by Permutational Multivariate Analysis of Variance (PERMANOVA) using the adonis function of the vegan package with default parameters [41]. Subsequently, the presence of statistically significant differences between groups of the different variables, as well as the presence of statistically significant differences in group dispersion, was evaluated using pairwise PERMANOVA and pairwise Permutational Multivariate Analysis of Dispersion (PERMDISP) [42], with the statistical significance threshold set at 0.05.

## 3. Results

### 3.1. Preservation Method Affects Total Microbial Counts and Balance Between Populations

Bacterial viability over the 12-month storage period differed among the three preservation methods (cryopreservation, refrigeration, and lyophilization) and between control and solarized soil samples.

**Figure 1:**
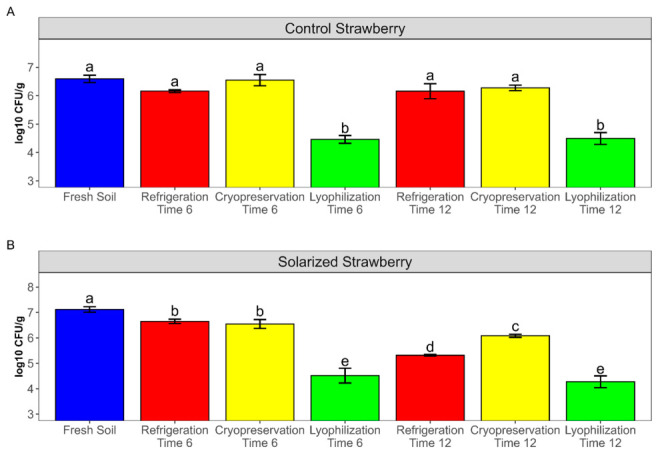
Total bacterial count in control (**A**) and solarized (**B**) soil samples under three different storage conditions, analyzed fresh and after 6 and 12 months. Data are presented as mean ± SD. Statistical differences were evaluated by performing one-way ANOVA analysis and Tukey Honestly Significant Difference (HSD) test for multiple comparisons across all storage methods and timepoints within soil type (Supplementary Tables S3 and S4). Different lowercase letters denote significant differences among groups within control and treated samples (threshold *p* < 0.05).

In control soil, bacterial abundance exhibited distinct preservation method-specific responses (Figure 1A; Supplementary Tables S3 and S4). After 6 months and 12 months of storage, cryopreserved and refrigerated samples maintained CFU g^−1^ levels comparable to those of fresh soil, whereas lyophilization caused significant reductions in bacterial viability (*p* < 0.001). In solarized soil, preservation challenges were more pronounced. Lyophilization exerted the most pronounced detrimental effects on bacterial viability at both 6 and 12 months (*p* < 0.05). Cryopreservation and refrigeration failed to maintain bacterial abundance at levels comparable to those of fresh soil after 6 and 12 months of storage (*p* < 0.05), resulting in substantial losses of viable bacterial populations, with the greatest reductions observed after 12 months (Figure 1B; Supplementary Tables S3 and S4).

In control soil, cryopreservation showed moderate efficiency, maintaining the oligotrophic fraction and causing only a limited reduction in copiotrophs from 6 months of storage onward (*p* < 0.01) (Table 1). Lyophilization caused pronounced reductions in both copiotrophic and oligotrophic populations at all timepoints (all *p* < 0.001). Refrigeration behaved similarly to cryopreservation, with a significant decline in the copiotrophic fraction after 6 months (*p* < 0.001) but no significant changes in oligotrophs. The EPi index showed no significant difference between groups when compared to fresh soils, highlighting stability in the distribution of bacterial populations. Minor changes were found in cryopreserved samples at 12 months of storage compared to fresh control soil, although no significant difference was highlighted. In solarized soil, cryopreservation outperformed the other preservation methods, with only a moderate decrease in oligotrophs after 12 months compared with fresh soil (*p* < 0.001) (Table 1). Lyophilization again led to substantial losses in both bacterial fractions at all storage times (all *p* < 0.001), confirming its limited suitability for preserving viable bacterial communities. Refrigeration caused an initial increase in copiotrophs after 6 months (*p* < 0.001), followed by a return to values comparable to fresh soil at 12 months, whereas the oligotrophic fraction declined at 12 months (*p* < 0.01). Initial fluctuation of the EPi index was highlighted for refrigerated samples at 6 months of storage compared to fresh solarized soil, but no statistical difference was found. No significant differences were found for all preservation methods in the EPi index compared to fresh solarized soils.

**Figure 2:**
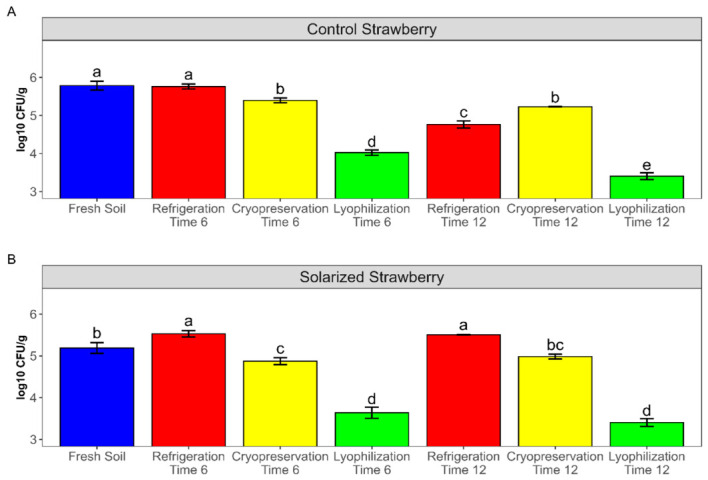

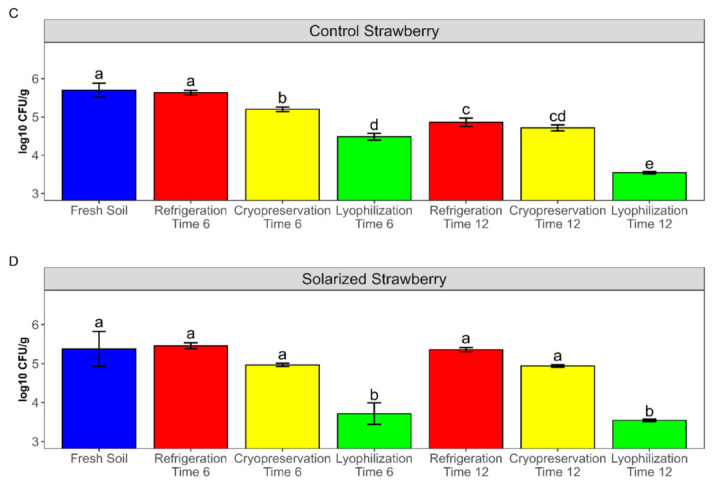
Total fungi count in control (**A**,**C**) and solarized (**B**,**D**) soil samples under three different storage conditions, fresh (Time 0) and after 6 and 12 months. Control and solarized samples were cultured on DRBCA (**A**,**B**) and MEA (**C**,**D**) media. Statistical differences between treatments were evaluated by performing one-way ANOVA analysis and Tukey Honestly Significant Difference (HSD) test for multiple comparisons across all storage methods and timepoints within soil type (Supplementary Tables S5 and S6). Data are presented as mean ± SD. Different lowercase letters denote significant differences between treatments within each soil type and growth medium analyzed (threshold *p* < 0.05).

Fungal viability was evaluated across three preservation methods (cryopreservation, lyophilization, refrigeration) over a 12-month storage period in control and solarized soils using two culture media (DRBCA and MEA). Lyophilization exerted the most pronounced detrimental effects on recoverable fungal CFU/g at both 6 and 12 months of storage across both soil types and culture media relative to fresh soil (all *p* < 0.001) (Figure 2A–D; Supplementary Tables S5 and S6). In control soil, cryopreservation resulted in moderate fungal losses compared to fresh soil at 6 months and 12 months of storage in both DRBCA (Figure 2A) and MEA media (Figure 2B) (*p* < 0.001). Refrigeration proved most effective for fungal recovery at 6 months across both media (Figure 2A,B), though significant reductions relative to fresh soil occurred by 12 months (all *p* < 0.001).

In solarized soil, refrigeration consistently maintains fungal yields across all timepoints in both DRBCA (Figure 2C) and MEA (Figure 2D) relative to fresh soil. Cryopreservation maintained CFU/g values comparable to fresh soil throughout the storage period when fungi were recovered in MEA medium; however, this trend was not observed on DRBCA, where a significant decrease was detected after 6 months of storage (*p* < 0.05), followed by a full recovery at 12 months of storage.

### 3.2. Functional Microbial Metabolic Profiles Differ by Preservation Method

The functional-level community profile of soil samples was assessed by examining carbon substrate utilization patterns using the BIOLOG EcoPlate™ assay (BIOLOG Inc., Hayward, CA, USA). Values of AWCD from each sample during incubation were monitored and plotted to establish the suitable end point of the analysis (Supplementary Figure S1 and Supplementary Table S7).

**Figure 3:**
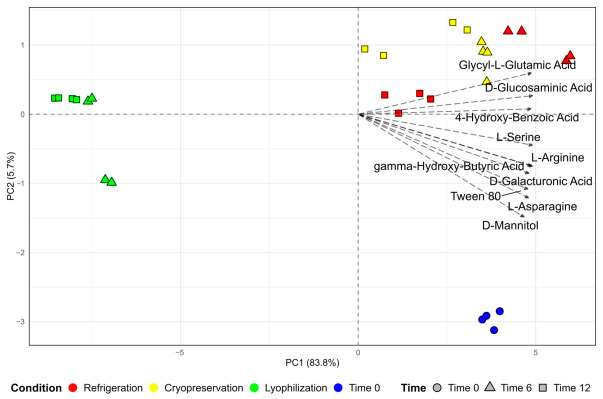
Principal Component Analysis (PCA) biplot displaying the metabolic profiles of microbial communities in control and solarized soils, fresh (time 0) and under different preservation methods (cryopreservation, lyophilization, and refrigeration) and storage durations (6 and 12 months). Points represent samples with colors indicating storage conditions and shapes denoting the analysis timepoint. Arrows represent the 10 most contributing carbon sources to the principal components in the graph. The first two components explain 83.8% and 5.7% of the variance, respectively. Each point corresponds to the metabolic profile of a sample measured at the timepoint of maximum color development (plateau) in the wells during incubation (Supplementary Figure S1 and Table S7).

In the PCA plot, Principal Component 1 (PC1) explains 83.8% of the total variance, indicating the presence of a strong dominant gradient in the dataset (Figure 3). This exceptionally high percentage of variance captured by a single axis is directly attributable to the severe catabolic suppression induced by lyophilization. Indeed, PC1 essentially acts as a strict gradient that separates the metabolically impaired freeze-dried samples from the active microbial communities of fresh, cryopreserved, and refrigerated soils. Clustering is primarily influenced by amino acid sources, such as L-Arginine and L-Serine, and by organic acids, such as gamma-Hydroxy-Butyric Acid and 4-Hydroxy-Benzoic Acid, which show strong associations with PC1 according to the directions of the related vectors in the plot (Supplementary Table S8). Samples under cryopreservation and refrigeration conditions, at both 6 and 12 months, cluster near the fresh soil controls (Time 0), suggesting some similarity in metabolic profiles among these groups. In contrast, lyophilized samples form a distinct cluster separated along PC1 and PC2, with PC2 accounting for 5.7% of the variance (Supplementary Table S9).

To better investigate the type of metabolite consumed by microbial communities, we quantified substrate utilization through optical density measurements (OD590) as an indicator of microbial catabolic activity in soil samples subjected to different storage methods over 12 months.

**Figure 4:**
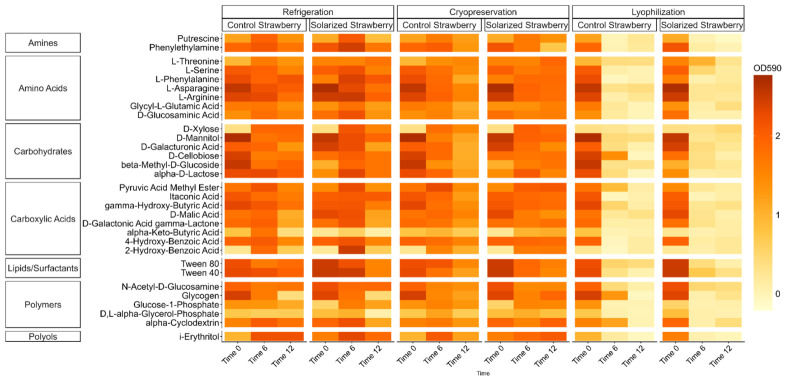
Heatmap of carbon consumption displaying the metabolic profiles of microbial communities in control and solarized soils, fresh (time 0) and under different preservation methods (cryopreservation, lyophilization, and refrigeration) and storage durations (6 and 12 months). On the *x*-axis are represented the timepoints of the analysis, and on the *y*-axis the carbon sources of BIOLOG EcoPlates. Each column represents the mean metabolic profile of two technical replicates for each soil sample measured at the timepoint of maximum color development (plateau) in the wells during incubation (Supplementary Figure S1 and Table S7).

Cryopreservation preserved amino acid, carboxylic acid, and lipid utilization relatively well in control soil (Figure 4); however, carbohydrate metabolism increased substantially during storage, with marked consumption of D-Xylose indicating selective utilization of pentose substrate. Loss of alpha-Cyclodextrin consumption suggested impaired polymer catabolism. In cryopreserved solarized soil (Figure 4), carbohydrate and amino acid metabolism showed greater metabolic resilience compared to control soil.

Heterogeneous residual substrate utilization persisted in lyophilized samples only in carboxylic acids and lipids, while amino acid consumption was largely eliminated except for selective substrates (L-Threonine, L-Asparagine, L-Arginine) and specific carbohydrates (D-Mannitol, beta-Methyl-D-Glucoside). Lyophilized solarized soil (Figure 4) showed similarly dramatic catabolic suppression.

Refrigeration maintained the highest metabolic activity across all substrate classes in control soil (Figure 4). Amine, amino acid, carbohydrate, and polyol utilization remained robust and well-preserved throughout storage. Notably, microbial communities displayed diminished consumption of specific carboxylic acids (gamma-Hydroxy-Butyric Acid and 4-Hydroxy-Benzoic Acid), suggesting metabolic reorientation toward alternative carbon sources or stress-responsive catabolic pathways. Glycogen consumption was notably reduced, indicating preferential catabolism of more readily available substrates. In refrigerated solarized soil (Figure 4), metabolic profiles remained similarly well-preserved, maintaining high utilization across metabolite classes comparable to control soil.

**Figure 5:**
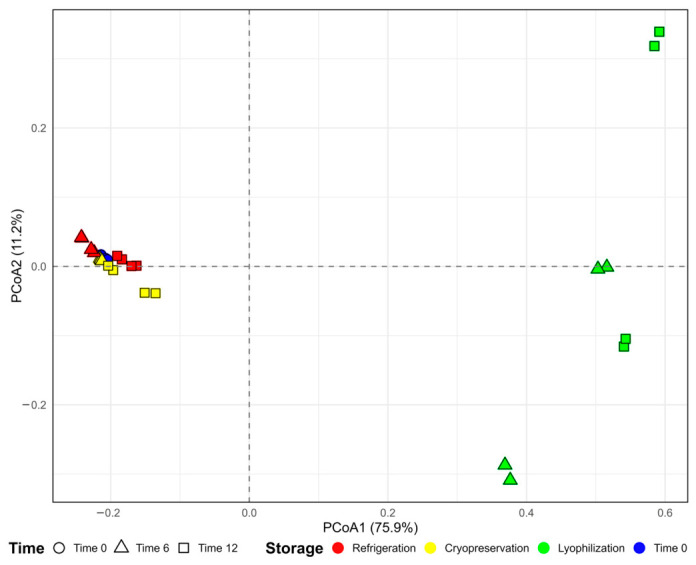
Principal Coordinate Analysis (PCoA) plot showing functional-level differentiation in soil microbial metabolic profiles among control and solarized soils, fresh (time 0) and under different preservation methods (cryopreservation, lyophilization, and refrigeration) and storage durations (6 and 12 months). The plot displays Bray–Curtis dissimilarity distances derived from BIOLOG EcoPlates carbon substrate utilization patterns. Dashed reference lines indicate the origin (0, 0) on both axes. Each point corresponds to the metabolic profile of a sample measured at the timepoint of maximum color development (plateau) in the wells during incubation (Supplementary Figure S1 and Table S7).

The functional-level community metabolic profile of soil microbial assemblages was assessed through Principal Coordinate Analysis (PCoA) based on Bray–Curtis dissimilarity distances calculated from carbon substrate utilization patterns obtained via the Biolog^®^ EcoPlate™ technique developed by Biolog Inc. in Hayward, CA, USA (Figure 5). PCoA analysis revealed notable clustering patterns that appeared to be associated with sample preservation methods and storage duration. Principal Coordinate 1 (PCoA1) accounted for 75.9% of the dataset variance, with samples tending to segregate along this axis according to preservation treatment. Cryopreserved and refrigerated samples at both 6 and 12 months of storage generally clustered in proximity to the fresh soil (Time 0), suggesting that these preservation methods may help retain aspects of the original functional metabolic profile. Lyophilized samples, however, showed apparent separation from the fresh soil and other preservation treatments along both PCoA1 and PCoA2 axes, potentially indicating alterations in metabolic phenotype following storage via this method. Principal Coordinate 2 (PCoA2) accounted for 11.2% of the variance and appeared to capture additional metabolic variation among samples. Temporal trends were observed within each preservation treatment, with Time 0 samples clustering near the origin, while samples stored for 6 and 12 months showed variable positioning depending on the preservation method.

### 3.3. Shifts in Microbial Community Structure over Time (16S and ITS)

Targeted amplicon sequencing analysis was performed to evaluate the effect of storage methods on soil microbial communities. Alpha-diversity metrics were calculated to assess microbial community structure and composition, including the Shannon diversity index, number of observed features, Pielou index of evenness and Simpson’s Diversity, while for beta diversity the Bray–Curtis Dissimilarity was used.

**Figure 6:**
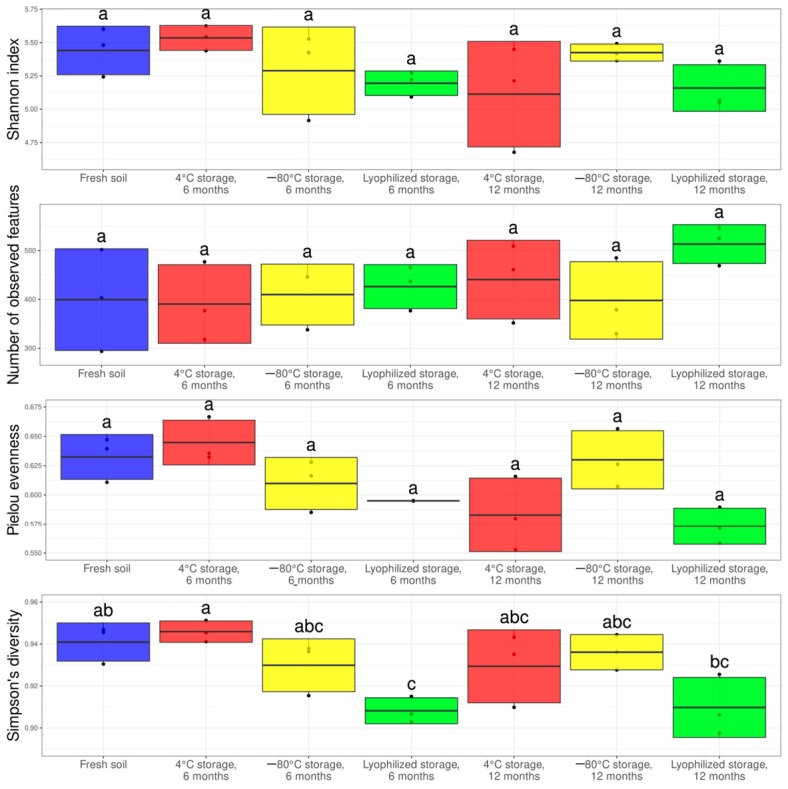
Boxplot-like plot associated with Shannon Index, number of observed features, Pielou evenness, and Simpson Diversity values measured for the fungal communities of solarized soil, grouped according to storage protocol and sampling timepoint. The middle line of each box corresponds to the mean of the distribution, while the upper and lower box bounds are set one standard deviation from the mean, and the whiskers extend to the minimum and maximum values, respectively. Letters above each box indicate the significance group, based on a one-way ANOVA test and a Tukey Honestly Significant Difference (HSD) test, with the significance threshold set at 0.05.

**Figure 7:**
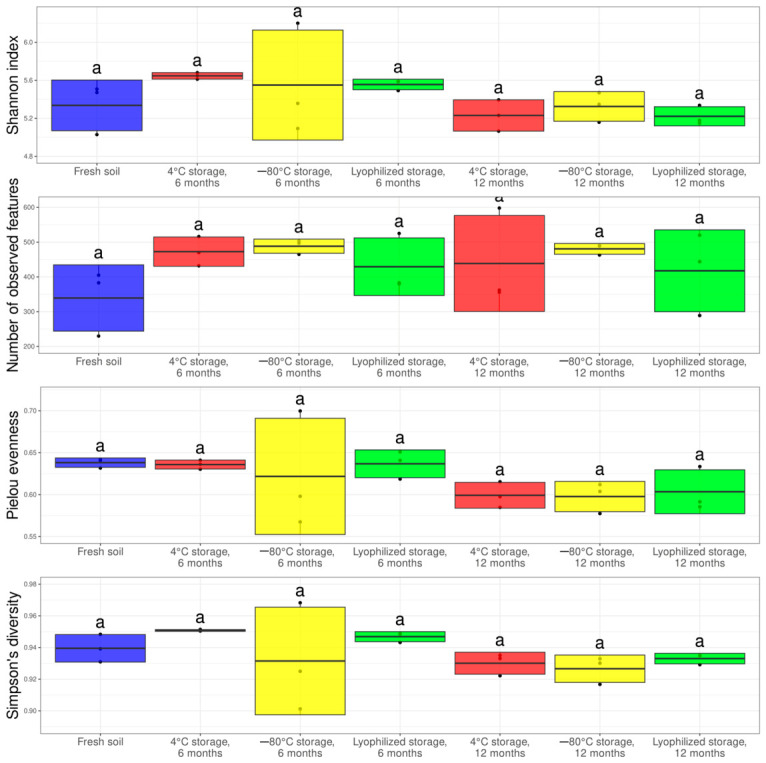
Boxplot-like plot associated with Shannon Index, number of observed features, Pielou evenness, and Simpson Diversity values measured for the fungal communities of control soil, grouped according to storage protocol and sampling timepoint. The middle line of each box corresponds to the mean of the distribution, while the upper and lower box bounds are set one standard deviation from the mean, and the whiskers extend to the minimum and maximum values, respectively. Letters above each box indicate the significance group, based on a one-way ANOVA test and a Tukey Honestly Significant Difference (HSD) test, with the significance threshold set at 0.05.

For the alpha diversity parameters associated with fungal communities, Figure 6 shows the results for the solarized soil, while Figure 7 shows the results for the control soil. In the solarized samples, no statistically significant difference was observed among the considered sampling timepoints and protocols for Shannon index, number of observed features, and Pielou evenness, while statistically significant differences were observed for Simpson diversity. In particular, soil collected at 6 months of storage from samples stored at 4 °C, as well as fresh soil, presented a significantly higher value (0.946 ± 0.005 and 0.941 ± 0.009, respectively) compared to lyophilized soil stored for 6 months (0.908 ± 0.006). In addition, soil collected at 6 months of storage from samples stored at 4 °C also presented significantly higher values compared to the lyophilized soil after 12 months of storage (0.910 ± 0.014). In contrast, no statistically significant difference was observed for any of the considered metrics in samples from the control soil.

**Figure 8:**
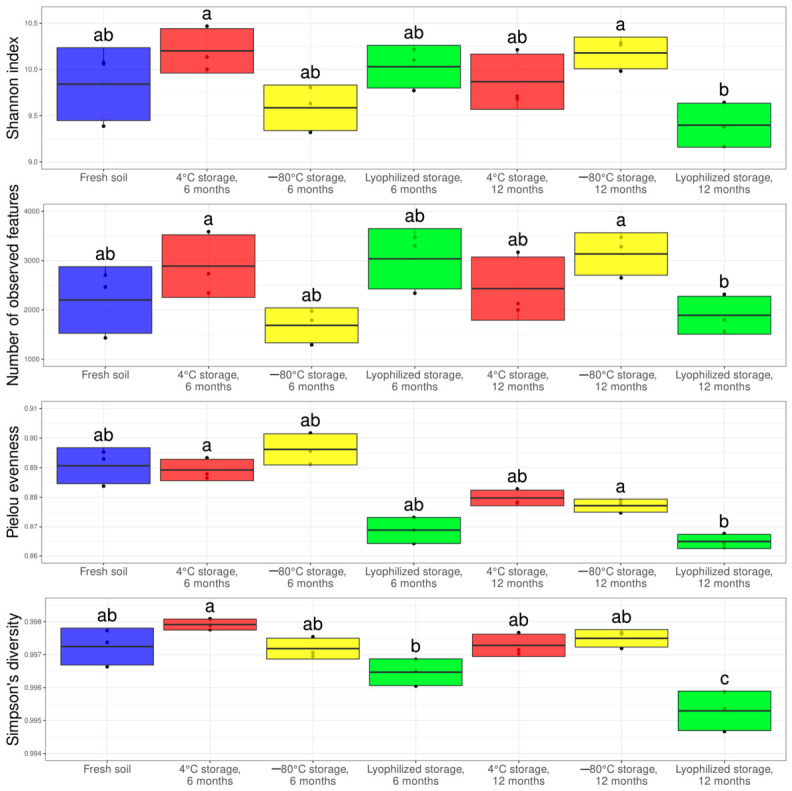
Boxplot-like plot associated with Shannon Index, number of observed features, Pielou evenness, and Simpson Diversity values measured for the bacterial communities of solarized soil, grouped according to storage protocol and sampling timepoint. The middle line of each box corresponds to the mean of the distribution, while the upper and lower box bounds are set one standard deviation from the mean, and the whiskers extend to the minimum and maximum values, respectively. Letters above each box indicate the significance group, based on a one-way ANOVA test and a Tukey Honestly Significant Difference (HSD) test, with the significance threshold set at 0.05.

**Figure 9:**
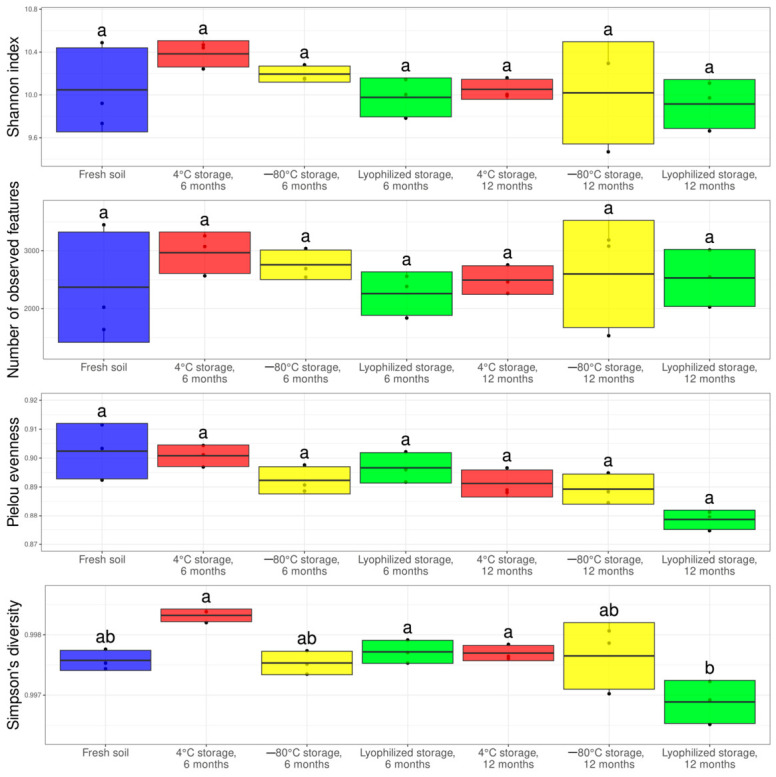
Boxplot-like plot associated with Shannon Index, number of observed features, Pielou evenness, and Simpson Diversity values measured for the bacterial communities of control soil, grouped according to storage protocol and sampling timepoint. The middle line of each box corresponds to the mean of the distribution, while the upper and lower box bounds are set one standard deviation from the mean, and the whiskers extend to the minimum and maximum values, respectively. Letters above each box indicate the significance group, based on a one-way ANOVA test and a Tukey Honestly Significant Difference (HSD) test, with the significance threshold set at 0.05.

For bacteria, Figure 8 shows the results for the solarized soil, while Figure 9 shows the results for the control soil. For the solarized soil, the presence of statistically significant differences was observed in all considered metrics. In particular, for the Shannon index, samples associated with soil stored at 4 °C for 6 months and soil stored at −80 °C for 12 months present significantly higher values (10.20 ± 0.24 and 10.18 ± 0.17, respectively) compared to lyophilized soil after 12 months of storage (9.40 ± 0.24). This pattern was also observed for the number of observed features (2887.67 ± 635.28 and 3134.00 ± 429.79, respectively, compared to 1891.00 ± 384.71) and Pielou evenness (0.89 ± 0.004 and 0.88 ± 0.002, respectively, compared to 0.86 ± 0.002). For the Simpson diversity, a different pattern emerged. In particular, lyophilized soil stored for 12 months presented a significantly lower value (0.995 ± 0.0006) compared to all other groups. Moreover, a statistically significant difference was observed between samples stored at 4 °C after 6 months of storage and lyophilized samples after 6 months of storage (0.997 ± 0.0002 and 0.996 ± 0.0004, respectively).

In contrast, for the control soil, the presence of statistically significant differences was observed only in the Simpson diversity. In particular, lyophilized soil samples stored for 12 months presented a significantly lower value (0.997 ± 0.0003) compared to samples stored at 4 °C, both after 6 months and 12 months of storage (0.998 ± 0.0001 and 0.998 ± 0.0001, respectively), as well as lyophilized soil samples after 6 months of storage (0.998 ± 0.0002).

For beta diversity, the results of the Adonis analysis for the fungal communities associated with solarized and control soil are presented in Table 2. In both the solarized soil and the control soil, the experimental group (defined as a combination of storage protocol and length of storage) presented a statistically significant effect on variance partitioning, with a slightly higher fraction in the solarized soil (R^2^ = 0.563, q-value = 0.001) compared to the control soil (R^2^ = 0.536, q-value = 0.001). While the results of the PCoA decomposition suggested the presence of differences (Supplementary Figure S2), pairwise PERMANOVA and PERMDISP comparisons of the data did not return any statistically significant differences between the considered groups (Supplementary Tables S10 and S11). This pattern suggests that the observed variation was distributed across multiple groups rather than driven by a single strong contrast, resulting in a significant overall effect but non-significant individual pairwise comparisons.

For the bacterial communities, the results of the Adonis analysis are presented in Table 3. For both control and solarized soils, similarly to what was observed for the fungal communities, the experimental group presented a statistically significant effect on variance partitioning, again with a larger fraction in the solarized soil (R^2^ = 0.646, q-value = 0.001) compared to the control soil (R^2^ = 0.577, q-value = 0.001). The PCoA ordination suggested separation among experimental groups, particularly according to storage protocol (Supplementary Figure S3), whereas pairwise PERMANOVA and PERMDISP analyses did not identify statistically significant differences between individual group pairs (Supplementary Tables S12 and S13).

### 3.4. Taxonomic Stability and Key Taxa Preservation

Genus-level analysis of bacterial and fungal communities was conducted on fresh and 12-month-old soil samples to assess whether preservation methods maintained genus-level stability despite potential class-level changes, thereby highlighting selective enrichment or depletion of specific bacterial and fungal lineages.

**Figure 10:**
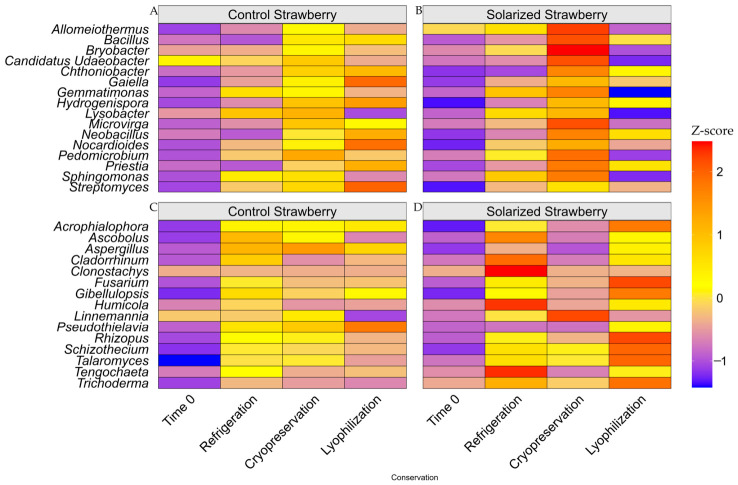
Heatmaps showing z-score-normalized abundance profiles of bacterial (**A**,**B**) and fungal (**C**,**D**) communities in control (**A**,**C**) and solarized (**B**,**D**) soil samples analyzed fresh and after 12 months of storage using the three different preservation methods. Genus-level taxa are displayed on the *y*-axis, with columns representing different preservation conditions (Time 0, Refrigeration, Cryopreservation, and Lyophilization). Z-scores standardize abundance values relative to the mean and standard deviation for each taxon, enabling direct comparison of relative changes across preservation methods.

In control samples, cryopreservation induced differences in relative abundances across most bacterial taxa compared to fresh soil (Time 0), except for *Candidatus Udaeobacter*, which maintained abundances similar to those in the initial community (Figure 10A, Supplementary Table S14). During lyophilization, the genera *Gaiella* and *Priestia* (all *p* ≤ 0.001) displayed changes in relative abundance. Refrigerated control samples showed bacterial genus composition largely similar to fresh soil, with significant differences restricted to *Gemmatimonas* and *Sphingomonas* (all *p* < 0.001), indicating that refrigeration induced differential shifts in select bacterial lineages. In solarized soils, cryopreservation induced shifts in relative abundances across all bacterial genera, displaying changes in *Bryobacter*, *Hydrogeniospora*, *Lysobacter*, and *Streptomyces* (all *p* < 0.05) (Figure 10B). During lyophilization, *Neobacillus*, *Priesta,* and *Streptomyces* (all *p* < 0.01) exhibited shifts in relative abundance. Refrigerated solarized samples displayed changes in abundances of *Bryobacter*, *Gemmatimonas*, *Lysobacter*, and *Sphingomonas* (all *p* < 0.05).

Concerning fungal communities, in control soil, cryopreservation induced changes in fungal genus composition compared to fresh soil (Time 0), with shifts in *Ascobulus* and *Aspergillus* (all *p* < 0.05) (Figure 10C, Supplementary Table S15). Lyophilized samples showed changes in the same taxa as cryopreserved samples relative to fresh soil, except for *Linnemannia*. Refrigeration induced similar compositional changes to cryopreservation but with greater intensity across multiple fungal genera. In solarized soils, cryopreservation maintained a similar fungal genus structure relative to fresh samples (Time 0), with changes restricted to *Linnemannia* (*p* < 0.05) (Figure 10D). Lyophilization induced changes in fungal genus abundances, with significant differences in *Fusarium*, *Gibellulopsis*, *Rhizopus*, and *Talaromyces* (all *p* < 0.05). Refrigeration also altered the original fungal community structure, accounting for shifts in relative abundance in *Ascobulus*, *Cladorrhinum*, *Fusarium*, *Gibellulopsis*, *Humicola*, and *Trichoderma* (all *p* < 0.05).

## 4. Discussion

Preserving soil microbiomes in biobanks is a key prerequisite for biodiversity conservation and bio-based solutions in sustainable agriculture [6,8]. While long-term storage is well established, the preservation of complex microbiomes remains challenging as storage conditions can profoundly alter microbial viability, community structure and functional potential [43]. Within the framework of the SUS-MIRRI.IT project (https://www.sus-mirri.it/it/; accessed on 19 November 2024), three preservation strategies—i.e., refrigeration at 4 °C, cryopreservation at −80 °C, and lyophilization—were evaluated over a 12-month period to assess their effectiveness in maintaining the stability and functionality of soil microbial consortia.

Cryopreservation, lyophilization, and refrigeration exerted distinct effects on the viability and balance of soil microbial communities. Storage at −80 °C is widely used for long-term microbiome preservation and generally provides better maintenance of microbial viability, though it can still influence community composition and functional traits in soils [4,13,44,45]. In the present study, cryopreservation at −80 °C proved to be the most effective method for preserving culturable bacterial populations in control soil, although it caused a moderate reduction in copiotrophs; it successfully maintained the oligotrophic fraction at levels comparable to fresh soil over the 12-month storage period, in line with previous studies [4,46,47]. However, this effect was not fully reproduced in solarized soil, where neither cryopreservation nor refrigeration maintained total bacterial counts after prolonged storage, indicating that the initial condition of the soil microbiome strongly influences its resilience to storage, with solarization increasing the susceptibility of the residual microbial community to preservation-induced stress. Importantly, cryopreservation still outperformed the other methods in this treated soil by keeping the copiotrophic fraction not significantly altered over the entire 12-month period. Although the EPi index did not differ significantly and remained stable throughout the storage period in both soils, indicating preservation of the overall eco-physiological balance between copiotrophic and oligotrophic bacteria, moderate reductions in specific bacterial populations suggest that cryopreservation at −80 °C may differentially affect microbial groups with distinct physiological traits. It is generally recognized that, during cryopreservation, only cells capable of tolerating freezing conditions are likely to remain viable. Consequently, in complex microbiome communities composed of multiple interacting populations, suboptimal cryopreservation may impose unintended selective pressures on the microbial population, reducing the recovery of more sensitive microorganisms while favoring the survival of freeze-tolerant cells [48]. This suggests that extreme freezing promotes selective survival rather than ensuring a completely neutral preservation of the soil microbiome [49,50].

Fungal communities responded differently from bacterial populations to the preservation methods tested, highlighting their greater sensitivity to storage conditions. Lyophilization consistently resulted in the greatest loss of fungal viability across both soil types, irrespective of the recovery medium, and was similarly detrimental to bacterial populations. These findings indicate that dehydration imposes strong selective pressure on complex soil microbiomes, substantially reducing the recovery of viable microorganisms. Although lyophilization is widely used for the long-term preservation of pure microbial cultures, it appears considerably less suitable for complex soil microbiomes, where it promotes selective survival and compromises the integrity of the native viable microbial community [11,12]. Cryopreservation generally provided better fungal preservation than lyophilization, but its performance depended on both soil condition and recovery medium. In control soil, fungal abundance progressively declined during storage, whereas in solarized soil, cryopreservation maintained fungal counts comparable to fresh soil on MEA throughout the 12-month period and only caused a transient reduction on DRBCA after six months, followed by complete recovery after 12 months. By contrast, refrigeration proved to be the most effective preservation strategy for fungi, maintaining fungal abundance throughout storage in solarized soil and providing the highest recovery in control soil during the first six months, although some decline was observed after prolonged storage. Overall, these results highlight the greater sensitivity of fungal communities to storage conditions and emphasize the need to consider different microbial functional groups when evaluating preservation strategies [51,52].

Functional-level analyses using BIOLOG EcoPlate assays demonstrated that preservation methods significantly influence microbial functional profiles. Both PCA and PCoA showed that cryopreserved and refrigerated samples clustered close to fresh soils after 6 and 12 months of storage, indicating that these preservation methods largely preserved the overall functional metabolic profile. In contrast, lyophilized samples formed a distinct cluster, reflecting the severe reduction in catabolic activity induced by dehydration, consistent with previous reports describing reduced microbial functional capacity and enzyme activity in dried soils [53]. Cryopreserved and refrigerated samples retained metabolic patterns similar to fresh soils, particularly for amino acid utilization, indicating preservation of key ecosystem functions. In contrast, lyophilization markedly altered metabolic profiles, especially reducing carbohydrate utilization, likely due to stress-induced loss of functional taxa [12]. Refrigeration provided the best overall preservation of metabolic activity, maintaining high substrate utilization across most carbon classes in both control and solarized soils. Our findings indicate that refrigeration effectively preserves the functional potential of soil microbiomes during medium-term storage, in agreement with previous reports describing limited functional alterations under refrigerated conditions. Overall, these findings demonstrate that storage conditions not only affect microbial abundance but also the functional potential of the soil microbiome, with important implications for interpreting post-storage metabolic assays [54].

Target metagenomic analyses showed that the response of soil microbial communities to preservation was strongly influenced by both the storage method and the initial condition of the microbiome. Alpha-diversity analyses further revealed that microbial responses to preservation stress were both taxon- and matrix-dependent. Fungal diversity remained relatively stable across treatments and timepoints, indicating a robust baseline community, although lyophilization reduced community evenness and increased dominance, particularly in solarized soils. Bacterial communities were more sensitive to preservation stress, particularly in solarized soils, where lyophilization significantly decreased diversity across multiple metrics. In contrast, cryopreservation and refrigeration maintained bacterial richness and diversity indices over time. These findings indicate that preservation methods have a stronger impact on bacterial diversity when the original microbiome has already been altered by environmental disturbance. Beta-diversity analyses further demonstrated that the preservation method was the major driver of microbial community differentiation after storage. Preserved samples progressively diverged according to the storage treatment, with lyophilized communities showing the greatest separation from the fresh baseline, indicating that dehydration exerts the strongest selective pressure on complex soil microbiomes. Over time, samples subjected to the same treatment clustered together, indicating reproducible preservation-driven trajectories. These results align with the literature showing that storage methods significantly affect microbial diversity, with freeze-drying among the most disruptive [5,12,55]. The genus-level analysis demonstrated that preservation effects were selective rather than uniform, revealing shifts in specific bacterial and fungal lineages even when broader taxonomic stability was retained. In bacterial communities, refrigeration best preserved the genus composition of control soils, whereas cryopreservation and lyophilization induced more pronounced changes in several taxa, suggesting differential sensitivity of bacterial genera to storage-related stress. In contrast, fungal communities appeared generally more responsive to preservation treatments, particularly under refrigeration and lyophilization, which altered multiple genera in both control and solarized soils. Notably, cryopreservation better maintained the fungal genus structure of solarized soils, with only limited variation relative to fresh samples, indicating that the impact of storage may depend not only on the preservation method but also on the initial soil condition. Overall, these findings suggest that preservation methods may still promote selective enrichment or depletion of taxa with distinct ecological or physiological traits, consistent with previous studies showing taxon-specific responses to storage conditions [5,12,14,46,52]. Taken together, these findings highlight that preservation-induced selective pressures are taxon-dependent and that bacterial and fungal communities respond differently to storage stress, with fungi generally more sensitive to lyophilization than bacteria [5]. Lyophilization may bias the results towards resistant lineages and underestimate functional diversity, whereas cryopreservation provides a more accurate representation of the fresh baseline sample microbial communities.

## 5. Limitations

This study has some methodological limitations that should be considered when interpreting the results. First, soil samples were processed without the addition of commonly used cryo- and lyoprotectants, which are known to improve cell survival and help maintain community structure and function during freezing and freeze-drying of microbial cultures and complex communities [55,56]. Our primary aim was to establish a baseline evaluation of preservation methods under minimally modified conditions that resemble simple procedures often applied in environmental and agricultural laboratories, where standardized cryoprotectant formulations are not always available [12,57]. Furthermore, including protective additives could have confounded the interpretation of the intrinsic effects of the preservation methods themselves, as these compounds may exert taxon-specific influences on microbial survival and community structure while substantially increasing experimental complexity [58]. In the present study, we did not include a positive control for the lyophilisation process (e.g., soils spiked with well-characterized reference strains). Exogenous microorganisms could introduce strain-specific responses and alter soil–microbe interactions, reducing the ecological relevance of the measurements. We acknowledge that this omission limits direct benchmarking of absolute process efficiency and reduces comparability with studies focused on methodological validation; however, it does not affect the internal comparative assessment of preservation treatments presented in this work. Concerning the experimental design, it was based on composite soil samples obtained by pooling soil samples from multiple plants within each plot. This approach, which provides a robust and homogeneous matrix for evaluating the relative impact of preservation methods across technical replicates, also limits the assessment of in-field biological variance and the broader generalizability of the solarization treatment effects.

## 6. Conclusions

Soil microbiome preservation is crucial for ensuring reproducibility and reliability in agronomic and environmental research. This study, integrating 16S/ITS metabarcoding, BIOLOG metabolic profiling, r/k strategists and microbial counts, provides clear evidence that community stability strongly depends on the storage method used, with important implications for microbiome research and the standardization of soil banking protocols. Cryopreservation emerged as the most effective strategy for maintaining overall microbial abundance (particularly in control soils), eco-physiological stability (e.g., preserving the copiotrophic fraction in solarized soils), genus-level stability and general metabolic function over a 12-month period. This preservation method best maintained overall catabolic profiles, although specific metabolic alterations were still observed depending on the storage duration. Refrigeration preserves moderate viability while maintaining robust metabolic activity and genus-level stability, making it suitable for short- to medium-term storage. In contrast, lyophilization, especially without lyoprotectants, imposed the greatest selective pressure, causing substantial losses in microbial viability and pronounced community shifts toward stress-tolerant taxa. Differences between bacterial and fungal responses highlight the need to account for storage-related biases when comparing microbiome data. These findings underscore the necessity of selecting preservation methods that minimize artificial shifts in microbiome composition. Collectively, this research provides a methodological baseline for the development of standardized long-term strategies for the conservation of agricultural soil biodiversity. By ensuring that preserved soil samples more faithfully represent baseline microbial conditions as similar as possible to those of the original sample collected, this work supports robust interpretations of soil microbiome studies and reliable downstream technical analyses.

## Figures and Tables

**Table 1:** Eco-physiological distribution—copiotrophs, oligotrophs counts and calculated EPi index—of bacterial populations in control and solarized soil samples under three different storage conditions, analyzed in fresh soil and after 6 and 12 months. Data were analyzed using one-way ANOVA with Tukey’s Honestly Significant Difference (HSD) test for multiple comparisons. For the EPi index, lowercase letters denote significant differences among treatments. For copiotrophs and oligotrophs, different capital letters denote significant differences among rows, while lowercase letters denote significant differences among treatments (threshold *p* < 0.05).

Condition	Time	Copiotrophs	Oligotrophs	EPi Index
Solarized Soil				
Fresh Soil	Time 0	6.27 × 10^5^ ± 2.54 × 10^5 aA^	1.51 × 10^6^ ± 1.41 × 10^5 aB^	0.558 ± 0.085 ^a^
Cryopreservation	Time 6 months	5.06 × 10^5^ ± 1.63 × 10^5 aA^	3.71 × 10^6^ ± 1.44 × 10^6 aB^	0.346 ± 0.047 ^a^
	Time 12 months	4.69 × 10^5^ ± 3.28 × 10^5 aA^	1.47 × 10^5^ ± 5.27 × 10^4 bA^	0.565 ± 0.104 ^a^
Lyophilization	Time 6 months	4.89 × 10^3^ ± 1.20 × 10^3 bA^	3.72 × 10^3^ ± 1.91 × 10^3 cA^	0.659 ± 0.054 ^a^
	Time 12 months	7.59 × 10^3^ ± 3.86 × 10^3 bA^	2.04 × 10^4^ ± 9.23 × 10^3 dA^	0.401 ± 0.261 ^a^
Refrigeration	Time 6 months	5.09 × 10^6^ ± 2.92 × 10^6 cA^	3.79 × 10^6^ ± 1.60 × 10^6 aA^	0.600 ± 0.121 ^a^
	Time 12 months	3.59 × 10^5^ ± 1.29 × 10^5 aA^	3.63 × 10^5^ ± 3.12 × 10^5 bA^	0.641 ± 0.054 ^a^
Control Soil				
Fresh Soil	Time 0	6.86 × 10^5^ ± 2.93 × 10^4 aA^	5.16 × 10^5^ ± 1.90 × 10^5 aA^	0.669 ± 0.035 ^a^
Cryopreservation	Time 6 months	4.95 × 10^4^ ± 1.59 × 10^4 bA^	3.17 × 10^5^ ± 1.76 × 10^5 aB^	0.422 ± 0.118 ^a^
	Time 12 months	6.71 × 10^4^ ± 4.03 × 10^4 bA^	1.27 × 10^5^ ± 8.39 × 10^4 aB^	0.469 ± 0.170 ^a^
Lyophilization	Time 6 months	2.49 × 10^2^ ± 8.43 × 10^1 dA^	1.62 × 10^2^ ± 1.44 × 10^2 bA^	0.629 ± 0.111 ^a^
	Time 12 months	1.76 × 10^1^ ± 1.22 × 10^1 cA^	4.90 × 10^1^ ± 3.35 × 10^1 bA^	0.659 ± 0.038 ^a^
Refrigeration	Time 6 months	3.29 × 10^4^ ± 2.70 × 10^4 bA^	1.29 × 10^5^ ± 3.54 × 10^4 aB^	0.541 ± 0.059 ^a^
	Time 12 months	6.35 × 10^4^ ± 5.56 × 10^4 bA^	1.46 × 10^5^ ± 8.27 × 10^4 aA^	0.539 ± 0.134 ^a^

**Table 2:** Results of the Adonis analysis for the fungal communities associated with the solarized and control soil samples. Df: degrees of freedom; SumOfSqs: sum of squares; MeanSqs: mean of squares. R2 is the total variance, expressed in decimal form, explained by the associated parameter, while the q-value is the false discovery rate (FDR) adjusted *p*-value using the Benjamini–Hochberg correction. The experimental group of a sample is the combination of storage protocol and length of storage for that sample. The significance threshold was set at 0.05. nd stands for “not defined”.

Parameter	Df	SumsOfSqs	MeanSqs	F.Model	R2	q-Value
Solarized soil						
Experimental group	6	0.545	0.091	3.006	0.563	0.001
Residuals	14	0.423	0.030	nd	0.437	nd
Total	20	0.968	nd	nd	1	nd
Control soil						
Experimental group	6	0.412	0.069	2.692	0.536	0.001
Residuals	14	0.357	0.025	nd	0.465	nd
Total	20	0.769	nd	nd	1	nd

**Table 3:** Results of the Adonis analysis for the bacterial communities associated with the solarized and control soil samples. Df: degrees of freedom; SumOfSqs: sum of squares; MeanSqs: mean of squares. R2 is the total variance, expressed in decimal form, explained by the associated parameter, while the q-value is the false discovery rate (FDR) adjusted *p*-value using the Benjamini–Hochberg correction. The experimental group of a sample is the combination of storage protocol and length of storage for that sample. The significance threshold was set at 0.05. nd stands for “not defined”.

Parameter	Df	SumsOfSqs	MeanSqs	F.Model	R2	q-Value
Solarized soil						
Experimental group	3	0.683	0.114	4.259	0.646	0.001
Residuals	14	0.374	0.027	nd	0.354	nd
Total	20	1.058	nd	nd	1	nd
Control soil						
Experimental group	3	0.586	0.098	3.190	0.577	0.001
Residuals	14	0.428	0.031	nd	0.422	nd
Total	20	1.014	nd	nd	1	nd

## Data Availability

Available BioProject PRJEB107956 in the National Center for Biotechnology Information (NCBI) “Sequence Read Archive” (SRA). Data will be made available upon request.

## Supplementary Materials

The following supporting information can be downloaded at https://www.mdpi.com/article/10.3390/microorganisms14071547/s1. Table S1: Raw and trimmed read count, indicated as number of read pairs, for fungal samples associated with control and solarized strawberry soil. m.s. = months of storage. Rep. = Replicate. Table S2: Raw and trimmed read count, indicated as number of read pairs, for bacterial samples associated with control and solarized strawberry soil. m.s. = months of storage. Rep. = Replicate. Table S3: One-way ANOVA results for bacterial abundance across experimental groups, consisting in the combination of preservation methods and storage time. Df: degrees of freedom; SumOfSqs: sum of squares; MeanSqs; mean of squares. F-value is the statistic’s value and the q-value is the false discovery rate (FDR) adjusted *p*-value using the Benjamini–Hochberg correction. Table S4: Pairwise comparisons of experimental groups, defined as combinations of preservation methods and storage time for bacterial abundances. *diff*: estimated difference between group means; *lwr* and *upr*: lower and upper bounds of the confidence interval, respectively; *p_value*: significance of the contrast (adjusted *p*-value, if applicable). “Contrast of Experimental Groups” indicates the specific pairwise comparison performed. Table S5: One-way ANOVA results for fungi abundance across preservation methods and storage time. Df: degrees of freedom; SumOfSqs: sum of squares; MeanSqs; mean of squares. F-value is the statistic’s value and the q-value is the false discovery rate (FDR) adjusted *p*-value using the Benjamini–Hochberg correction. Table S6: Pairwise comparisons of experimental groups, defined as combinations of preservation methods and storage time for fungi abundances in different culture media. *diff*: estimated difference between group means; *lwr* and *upr*: lower and upper bounds of the confidence interval, respectively; *p_value*: significance of the contrast (adjusted *p*-value, if applicable). “Contrast of Experimental Groups” indicates the specific pairwise comparison performed. Table S7: Max AWCD for each soil sample during incubation in the BIOLOG EcoPlates analysis. Table S8: Loadings of preservation method-sample combinations on principal components PC1 and PC2 of Figure 3 in the main manuscript. Table S9: Loadings of metabolites on principal components PC1 and PC2 of Figure 3 in the main manuscript. Table S10: PERMANOVA and PERMDISP analysis results for fungal communities associated with (solarized) strawberry soil samples. Pseudo-F and F-value indicate the statistic measured by PERMANOVA and PERMDISP, respectively, while the q-value is the false discovery rate (FDR) adjusted *p*-value. Significance threshold was set at 0.05. Table S11: PERMANOVA and PERMDISP analysis results for fungal communities associated with (control) strawberry soil samples. Pseudo-F and F-value indicate the statistic measured by PERMANOVA and PERMDISP, respectively, while the q-value is the false discovery rate (FDR) adjusted *p*-value. Significance threshold was set at 0.05. Table S12: PERMANOVA and PERMDISP analysis results for bacterial communities associated with (solarized) strawberry soil samples. Pseudo-F and F-value indicate the statistic measured by PERMANOVA and PERMDISP, respectively, while the q-value is the false discovery rate (FDR) adjusted *p*-value. Significance threshold was set at 0.05. Table S13: PERMANOVA and PERMDISP analysis results for bacterial communities associated with (control) strawberry soil samples. Pseudo-F and F-value indicate the statistic measured by PERMANOVA and PERMDISP, respectively, while the q-value is the false discovery rate (FDR) adjusted *p*-value. Significance threshold was set at 0.05. Table S14. Pairwise *p*-values from the genus-level differential abundance analysis performed using ANCOM-BC2. Rows represent bacterial genera, and columns report the *p*-values associated with the pairwise contrasts among the experimental groups defined by preservation treatment, storage time, and soil condition. Table S15. Pairwise *p*-values from the genus-level differential abundance analysis performed using ANCOM-BC2. Rows represent fungal genera, and columns report the *p*-values associated with the pairwise contrasts among the experimental groups defined by preservation treatment, storage time, and soil condition. Figure S1: AWCD curves of BIOLOG EcoPlates of soil samples during incubation. Figure S2: PCoA plot of fungal communities associated with (solarized) (A) and (control) (B) strawberry soil. Individual points represent samples, which are associated with a storage protocol (color) and sampling timepoints (shape). PC1: principal coordinate 1; PC2: principal coordinate 2. Percentage values for each axis indicate the total variance explained by each principal coordinate. Figure S3: PCoA plot of bacterial communities associated with (solarized) (A) and (control) (B) strawberry soil. Individual points represent samples, which are associated with a storage protocol (color) and sampling timepoints (shape). PC1: principal coordinate 1; PC2: principal coordinate 2. Percentage values for each axis indicate the total variance explained by each principal coordinate.
