# Supplementary material for: Assessment of Stability of Preservation Techniques for Strawberry Soil Microbiomes: Exploring the Biotechnological Relevance for Sustainable Agriculture
Source: Microorganisms. 2026 Jul 15;14(7):1547. doi: 10.3390/microorganisms14071547 (PMC13414444; doi:10.3390/microorganisms14071547)
Supplement: Supplementary file 1 [file microorganisms-14-01547-s001.zip › microorganisms-4194221-supplementary.pdf]

SUPPLEMENTARY TABLES

Table S1: raw and trimmed read count, indicated as number of read pairs, for fungal samples associated with control and solarized strawberry soil. m.s. = months of storage. Rep. = Replicate

	Control strawberry					
	**Raw reads (number of read pairs)**			**Trimmed reads (number of read pairs)**		
**Group**	**Rep. A**	**Rep. B**	**Rep. C**	**Rep. A**	**Rep. B**	**Rep. C**
Fresh soil	180751	72791	199851	179938	72095	198957
Refrigerated – 6 m.s.	441183	332341	375049	439105	330896	373141
Cryopreserved – 6 m.s.	283038	328176	420157	281689	326768	418060
Lyophilized – 6 m.s.	203369	463002	191048	201990	460810	190183
Refrigerated – 12 m.s.	340220	230085	920074	338169	228722	913633
Cryopreserved – 12 m.s.	342670	358814	632646	339252	356330	628029
Lyophilized – 12 m.s.	520630	371908	130619	517120	369606	129786
	**Solarized strawberry**					
Fresh soil	426986	216226	170136	425203	215011	169396
Refrigerated – 6 m.s.	130193	243473	430259	129500	242389	428244
Cryopreserved – 6 m.s.	263816	264770	283744	262581	263495	282573
Lyophilized – 6 m.s.	364309	360785	198600	362746	358862	197619
Refrigerated – 12 m.s.	575730	835491	339502	571564	830403	337355
Cryopreserved – 12 m.s.	484287	165287	217590	481430	164310	216337
Lyophilized – 12 m.s.	762993	587317	632053	758143	583002	627638

Table S2: raw and trimmed read count, indicated as number of read pairs, for bacterial samples associated with control and solarized strawberry soil. m.s. = months of storage. Rep. = Replicate

	Control strawberry					
	Raw reads (number of read pairs)			Trimmed reads (number of read pairs)		
**Group**	**Rep. A**	**Rep. B**	**Rep. C**	**Rep. A**	**Rep. B**	**Rep. C**
Fresh soil	132844	182759	447063	131224	180107	441692
Refrigerated – 6 m.s.	328008	256084	369473	323677	253132	365287
Cryopreserved – 6 m.s.	361077	260450	287919	356956	257274	284694
Lyophilized – 6 m.s.	298797	323639	178488	295405	320084	176410
Refrigerated – 12 m.s.	404762	373984	348326	398293	368019	342622
Cryopreserved – 12 m.s.	667389	656053	207416	657007	645670	204013
Lyophilized – 12 m.s.	348061	647708	365154	342391	637864	359419
	**Solarized strawberry**					
Fresh soil	312243	260758	121624	308477	257672	120203
Refrigerated – 6 m.s.	257433	432217	318225	254431	427065	313692
Cryopreserved – 6 m.s.	108689	200627	166134	107044	164260	164260
Lyophilized – 6 m.s.	290895	650126	684818	287672	642641	676492
Refrigerated – 12 m.s.	612515	314427	271025	602811	309306	266427
Cryopreserved – 12 m.s.	398709	763325	755207	392570	751594	743661
Lyophilized – 12 m.s.	216324	426352	277313	212734	419650	272592

Table S3. One-way ANOVA results for bacterial abundance across experimental groups, consisting in the combination of preservation methods and storage time. Df: degrees of freedom; SumOfSqs: sum of squares; MeanSqs; mean of squares. F-value is the statistic's value and the q-value is the false discovery rate (FDR) adjusted p-value using the Benjamini-Hochberg correction.

**Control Strawberry**						
	**Df**	**Sum Sq**	**Mean Sq**	**F value**	**q-value**	
Experimental Group	6	15,54326	2,590543	90,24706	2,22E-10	***
Residual	14	0,40187	0,028705			
**Solarized Strawberry**						
	**Df**	**Sum Sq**	**Mean Sq**	**F value**	**q-value**	
Experimental Group	6	21,91766	3,652944	133,3792	1,55E-11	***
Residual	14	0,383427	0,027388			

Table S4. Pairwise comparisons of experimental groups, defined as combinations of preservation methods and storage time for bacterial abundances. *diff*: estimated difference between group means; *lwr* and *upr*: lower and upper bounds of the confidence interval, respectively; *p_value*: significance of the contrast (adjusted p-value, if applicable). "Contrast of Experimental Groups" indicates the specific pairwise comparison performed.

**Control Strawberry**				
**diff**	**lwr**	**upr**	**p_value**	**Contrast of Experimental Group**
0.271715	-0.20064	0.744073	0.474865257	Cryopreservation_Time 6-Cryopreservation_Time 12
-1.78405	-2.25641	-1.31169	6.36033E-08	Lyophilization_Time 12-Cryopreservation_Time 12
-1.81803	-2.29039	-1.34568	4.97904E-08	Lyophilization_Time 6-Cryopreservation_Time 12
-0.11975	-0.59211	0.352608	0.972227789	Refrigeration_Time 12-Cryopreservation_Time 12
-0.11726	-0.58962	0.355096	0.974902419	Refrigeration_Time 6-Cryopreservation_Time 12
0.316921	-0.15544	0.789279	0.312253459	Fresh Soil-Cryopreservation_Time 12
-2.05576	-2.52812	-1.58341	9.71297E-09	Lyophilization_Time 12-Cryopreservation_Time 6
-2.08975	-2.56211	-1.61739	7.77447E-09	Lyophilization_Time 6-Cryopreservation_Time 6
-0.39146	-0.86382	0.080893	0.1368614	Refrigeration_Time 12-Cryopreservation_Time 6
-0.38898	-0.86134	0.083381	0.140961842	Refrigeration_Time 6-Cryopreservation_Time 6
0.045206	-0.42715	0.517564	0.999861427	Fresh Soil-Cryopreservation_Time 6
-0.03398	-0.50634	0.438373	0.999973914	Lyophilization_Time 6-Lyophilization_Time 12
1.6643	1.191942	2.136658	1.55067E-07	Refrigeration_Time 12-Lyophilization_Time 12
1.666788	1.19443	2.139146	1.52146E-07	Refrigeration_Time 6-Lyophilization_Time 12
2.100971	1.628613	2.573329	7.22854E-09	Fresh Soil-Lyophilization_Time 12
1.698285	1.225927	2.170643	1.19823E-07	Refrigeration_Time 12-Lyophilization_Time 6
1.700773	1.228414	2.173131	1.17602E-07	Refrigeration_Time 6-Lyophilization_Time 6
2.134956	1.662598	2.607314	5.81135E-09	Fresh Soil-Lyophilization_Time 6
0.002488	-0.46987	0.474846	1	Refrigeration_Time 6-Refrigeration_Time 12
0.436671	-0.03569	0.909029	0.07870998	Fresh Soil-Refrigeration_Time 12
0.434183	-0.03817	0.906541	0.081201048	Fresh Soil-Refrigeration_Time 6
**Solarized Strawberry**				
**diff**	**lwr**	**upr**	**p_value**	**Contrast of Experimental Group**
0.463427	0.002035	0.924819	0.048676649	Cryopreservation_Time 6-Cryopreservation_Time 12
-1.80896	-2.27035	-1.34757	3.91153E-08	Lyophilization_Time 12-Cryopreservation_Time 12
-1.56671	-2.0281	-1.10532	2.47777E-07	Lyophilization_Time 6-Cryopreservation_Time 12
-0.76744	-1.22883	-0.30605	0.000866494	Refrigeration_Time 12-Cryopreservation_Time 12
0.565753	0.104361	1.027145	0.012345015	Refrigeration_Time 6-Cryopreservation_Time 12
1.035509	0.574117	1.496901	3.66251E-05	Fresh Soil-Cryopreservation_Time 12
-2.27239	-2.73378	-1.811	1.83723E-09	Lyophilization_Time 12-Cryopreservation_Time 6
-2.03014	-2.49153	-1.56875	8.37211E-09	Lyophilization_Time 6-Cryopreservation_Time 6
-1.23087	-1.69226	-0.76948	4.87901E-06	Refrigeration_Time 12-Cryopreservation_Time 6
0.102326	-0.35907	0.563718	0.985641335	Refrigeration_Time 6-Cryopreservation_Time 6
0.572082	0.11069	1.033474	0.011334901	Fresh Soil-Cryopreservation_Time 6
0.242248	-0.21914	0.70364	0.572529672	Lyophilization_Time 6-Lyophilization_Time 12
1.041518	0.580126	1.502909	3.43028E-05	Refrigeration_Time 12-Lyophilization_Time 12
2.374713	1.913321	2.836105	1.04425E-09	Refrigeration_Time 6-Lyophilization_Time 12
2.844469	2.383077	3.305861	1.13946E-10	Fresh Soil-Lyophilization_Time 12
0.799269	0.337877	1.260661	0.000580713	Refrigeration_Time 12-Lyophilization_Time 6
2.132465	1.671073	2.593857	4.2919E-09	Refrigeration_Time 6-Lyophilization_Time 6

2.60222	2.140829	3.063612	3.4241E-10	Fresh Soil-Lyophilization_Time 6
1.333196	0.871804	1.794588	1.85262E-06	Refrigeration_Time 6-Refrigeration_Time 12
1.802951	1.341559	2.264343	4.08562E-08	Fresh Soil-Refrigeration_Time 12
0.469755	0.008364	0.931147	0.044772497	Fresh Soil-Refrigeration_Time 6

Table S5. One-way ANOVA results for fungi abundance across preservation methods and storage time. Df: degrees of freedom; SumOfSqs: sum of squares; MeanSqs; mean of squares. F-value is the statistic's value and the q-value is the false discovery rate (FDR) adjusted p-value using the Benjamini-Hochberg correction.

			**Control Strawberry DRBCA**			
	**Df**	**Sum Sq**	**Mean Sq**	**F value**	**q-value**	
Experimental group	6	14.71249	2.452081	396.5489	8.35E-15	***
Residual	14	0.08657	0.006184			
			**Solarized Strawberri DRBCA**			
	**Df**	**Sum Sq**	**Mean Sq**	**F value**	**q-value**	
Experimental group	6	13.4333	2.238884	263.4602	1.42E-13	***
Residual	14	0.118972	0.008498			
			**Control Strawberry MEA**			
	**Df**	**Sum Sq**	**Mean Sq**	**F value**	**q-value**	
Experimental group	6	9.957175	1.659529	172.2829	2.68E-12	***
Residual	14	0.134856	0.009633			
			**Solarized Strawberri MEA**			
	**Df**	**Sum Sq**	**Mean Sq**	**F value**	**q-value**	
Experimental group	6	11.62598	1.937664	46.60867	1.85E-08	***
Residual	14	0.582023	0.041573			

Table S6. Pairwise comparisons of experimental groups, defined as combinations of preservation methods and storage time for fungi abundances in different culture media. *diff*: estimated difference between group means; *lwr* and *upr*: lower and upper bounds of the confidence interval, respectively; *p_value*: significance of the contrast (adjusted p-value, if applicable). "Contrast of Experimental Groups" indicates the specific pairwise comparison performed.

**Control Strawberry DRBCA**				
**diff**	**lwr**	**upr**	**p_value**	**Contrast of Experimental Group**
0.167572	-0.05166	0.386808	0.194729	Cryopreservation_Time 6-Cryopreservation_Time 12
-1.82483	-2.04407	-1.6056	7.62E-13	Lyophilization_Time 12-Cryopreservation_Time 12
-1.20613	-1.42537	-0.98689	4.62E-10	Lyophilization_Time 6-Cryopreservation_Time 12
-0.46485	-0.68408	-0.24561	6.91E-05	Refrigeration_Time 12-Cryopreservation_Time 12
0.532228	0.312992	0.751464	1.49E-05	Refrigeration_Time 6-Cryopreservation_Time 12
0.555797	0.336561	0.775033	8.95E-06	Time 0_Time 0-Cryopreservation_Time 12
-1.9924	-2.21164	-1.77317	1.85E-13	Lyophilization_Time 12-Cryopreservation_Time 6
-1.3737	-1.59294	-1.15447	9.21E-11	Lyophilization_Time 6-Cryopreservation_Time 6
-0.63242	-0.85165	-0.41318	1.89E-06	Refrigeration_Time 12-Cryopreservation_Time 6
0.364656	0.14542	0.583891	0.000867	Refrigeration_Time 6-Cryopreservation_Time 6
0.388224	0.168989	0.60746	0.000466	Time 0_Time 0-Cryopreservation_Time 6
0.6187	0.399464	0.837936	2.47E-06	Lyophilization_Time 6-Lyophilization_Time 12
1.359985	1.140749	1.579221	1.05E-10	Refrigeration_Time 12-Lyophilization_Time 12
2.357059	2.137823	2.576294	6.59E-14	Refrigeration_Time 6-Lyophilization_Time 12
2.380628	2.161392	2.599863	6.36E-14	Time 0_Time 0-Lyophilization_Time 12
0.741285	0.522049	0.960521	2.61E-07	Refrigeration_Time 12-Lyophilization_Time 6
1.738359	1.519123	1.957594	1.95E-12	Refrigeration_Time 6-Lyophilization_Time 6
1.761928	1.542692	1.981163	1.5E-12	Time 0_Time 0-Lyophilization_Time 6
0.997074	0.777838	1.21631	5.34E-09	Refrigeration_Time 6-Refrigeration_Time 12
1.020643	0.801407	1.239879	3.89E-09	Time 0_Time 0-Refrigeration_Time 12
0.023569	-0.19567	0.242805	0.999728	Time 0_Time 0-Refrigeration_Time 6
**Solarized Strawberry DRBCA**				
**diff**	**lwr**	**upr**	**p_value**	**Contrast of Experimental Group**
-0.11006	-0.36707	0.146949	0.761111	Cryopreservation_Time 6-Cryopreservation_Time 12
-1.57915	-1.83616	-1.32214	1.19E-10	Lyophilization_Time 12-Cryopreservation_Time 12
-1.34516	-1.60218	-1.08815	8.47E-10	Lyophilization_Time 6-Cryopreservation_Time 12
0.519229	0.262219	0.77624	0.000117	Refrigeration_Time 12-Cryopreservation_Time 12
0.544391	0.287381	0.801401	6.99E-05	Refrigeration_Time 6-Cryopreservation_Time 12
0.204881	-0.05213	0.461892	0.163075	Time 0_Time 0-Cryopreservation_Time 12
-1.46909	-1.7261	-1.21208	2.91E-10	Lyophilization_Time 12-Cryopreservation_Time 6
-1.2351	-1.49211	-0.97809	2.54E-09	Lyophilization_Time 6-Cryopreservation_Time 6
0.629291	0.37228	0.886301	1.35E-05	Refrigeration_Time 12-Cryopreservation_Time 6
0.654452	0.397442	0.911463	8.49E-06	Refrigeration_Time 6-Cryopreservation_Time 6
0.314943	0.057932	0.571953	0.012405	Time 0_Time 0-Cryopreservation_Time 6
0.233988	-0.02302	0.490998	0.085507	Lyophilization_Time 6-Lyophilization_Time 12
2.098382	1.841371	2.355392	1.1E-12	Refrigeration_Time 12-Lyophilization_Time 12
2.123544	1.866533	2.380554	8.78E-13	Refrigeration_Time 6-Lyophilization_Time 12
1.784034	1.527024	2.041044	2.08E-11	Time 0_Time 0-Lyophilization_Time 12
1.864394	1.607384	2.121404	1E-11	Refrigeration_Time 12-Lyophilization_Time 6

1.889556	1.632545	2.146566	7.94E-12	Refrigeration_Time 6-Lyophilization_Time 6
1.550046	1.293036	1.807057	1.51E-10	Time 0_Time 0-Lyophilization_Time 6
0.025162	-0.23185	0.282172	0.999842	Refrigeration_Time 6-Refrigeration_Time 12
-0.31435	-0.57136	-0.05734	0.012585	Time 0_Time 0-Refrigeration_Time 12
-0.33951	-0.59652	-0.0825	0.006851	Time 0_Time 0-Refrigeration_Time 6
**Control Strawberry MEA**				
**diff**	**lwr**	**upr**	**p_value**	**Contrast of Experimental Group**
0.485779	0.212149	0.759409	0.000454	Cryopreservation_Time 6-Cryopreservation_Time 12
-1.17219	-1.44582	-0.89856	1.2E-08	Lyophilization_Time 12-Cryopreservation_Time 12
-0.2326	-0.50623	0.041033	0.121329	Lyophilization_Time 6-Cryopreservation_Time 12
0.145666	-0.12796	0.419296	0.558026	Refrigeration_Time 12-Cryopreservation_Time 12
0.918155	0.644525	1.191785	2.88E-07	Refrigeration_Time 6-Cryopreservation_Time 12
0.986726	0.713096	1.260356	1.15E-07	Time 0_Time 0-Cryopreservation_Time 12
-1.65797	-1.9316	-1.38434	1.42E-10	Lyophilization_Time 12-Cryopreservation_Time 6
-0.71838	-0.99201	-0.44475	5.91E-06	Lyophilization_Time 6-Cryopreservation_Time 6
-0.34011	-0.61374	-0.06648	0.011121	Refrigeration_Time 12-Cryopreservation_Time 6
0.432376	0.158746	0.706006	0.001415	Refrigeration_Time 6-Cryopreservation_Time 6
0.500947	0.227317	0.774577	0.000332	Time 0_Time 0-Cryopreservation_Time 6
0.939593	0.665963	1.213223	2.15E-07	Lyophilization_Time 6-Lyophilization_Time 12
1.317856	1.044226	1.591486	2.47E-09	Refrigeration_Time 12-Lyophilization_Time 12
2.090346	1.816716	2.363976	3.96E-12	Refrigeration_Time 6-Lyophilization_Time 12
2.158917	1.885287	2.432547	2.14E-12	Time 0_Time 0-Lyophilization_Time 12
0.378263	0.104633	0.651893	0.00469	Refrigeration_Time 12-Lyophilization_Time 6
1.150752	0.877122	1.424383	1.54E-08	Refrigeration_Time 6-Lyophilization_Time 6
1.219324	0.945693	1.492954	7.05E-09	Time 0_Time 0-Lyophilization_Time 6
0.772489	0.498859	1.046119	2.46E-06	Refrigeration_Time 6-Refrigeration_Time 12
0.84106	0.56743	1.114691	8.64E-07	Time 0_Time 0-Refrigeration_Time 12
0.068571	-0.20506	0.342201	0.973733	Time 0_Time 0-Refrigeration_Time 6
**Solarized Strawberry MEA**				
**diff**	**lwr**	**upr**	**p_value**	Contrast of Experimental Group
0.025958	-0.5425	0.594416	0.999998	Cryopreservation_Time 6-Cryopreservation_Time 12
-1.39771	-1.96617	-0.82925	1.28E-05	Lyophilization_Time 12-Cryopreservation_Time 12
-1.22505	-1.7935	-0.65659	5.77E-05	Lyophilization_Time 6-Cryopreservation_Time 12
0.41814	-0.15032	0.986598	0.226472	Refrigeration_Time 12-Cryopreservation_Time 12
0.519285	-0.04917	1.087743	0.083971	Refrigeration_Time 6-Cryopreservation_Time 12
0.435617	-0.13284	1.004075	0.192699	Time 0_Time 0-Cryopreservation_Time 12
-1.42367	-1.99213	-0.85521	1.03E-05	Lyophilization_Time 12-Cryopreservation_Time 6
-1.251	-1.81946	-0.68255	4.57E-05	Lyophilization_Time 6-Cryopreservation_Time 6
0.392182	-0.17628	0.96064	0.28496	Refrigeration_Time 12-Cryopreservation_Time 6
0.493327	-0.07513	1.061785	0.109576	Refrigeration_Time 6-Cryopreservation_Time 6
0.409659	-0.1588	0.978117	0.244467	Time 0_Time 0-Cryopreservation_Time 6
0.172665	-0.39579	0.741123	0.936261	Lyophilization_Time 6-Lyophilization_Time 12
1.815851	1.247393	2.384309	5.35E-07	Refrigeration_Time 12-Lyophilization_Time 12
1.916996	1.348538	2.485454	2.7E-07	Refrigeration_Time 6-Lyophilization_Time 12
1.833328	1.26487	2.401786	4.74E-07	Time 0_Time 0-Lyophilization_Time 12

1.643186	1.074728	2.211644	1.84E-06	Refrigeration_Time 12-Lyophilization_Time 6
1.744331	1.175873	2.312789	8.82E-07	Refrigeration_Time 6-Lyophilization_Time 6
1.660663	1.092205	2.229121	1.62E-06	Time 0_Time 0-Lyophilization_Time 6
0.101145	-0.46731	0.669603	0.995454	Refrigeration_Time 6-Refrigeration_Time 12
0.017477	-0.55098	0.585935	1	Time 0_Time 0-Refrigeration_Time 12
-0.08367	-0.65213	0.48479	0.998389	Time 0_Time 0-Refrigeration_Time 6

Table S7. Max AWCD for each soil sample during incubation in the BIOLOG EcoPlates analysis.

Storage	Time	Sample	Incubation Day	Rep	AWCD
Cryopreservation	Time 12	Control Strawberry	6	1	1.29699
Cryopreservation	Time 12	Control Strawberry	6	2	1.22717
Cryopreservation	Time 12	Solarized Strawberry	6	1	1.550437
Cryopreservation	Time 12	Solarized Strawberry	6	2	1.60309
Cryopreservation	Time 6	Control Strawberry	5	1	1.661979
Cryopreservation	Time 6	Control Strawberry	5	2	1.675512
Cryopreservation	Time 6	Solarized Strawberry	2	1	1.684167
Cryopreservation	Time 6	Solarized Strawberry	6	2	1.659237
Lyophilization	Time 12	Control Strawberry	6	1	0.116646
Lyophilization	Time 12	Control Strawberry	6	2	0.127692
Lyophilization	Time 12	Solarized Strawberry	5	1	0.178427
Lyophilization	Time 12	Solarized Strawberry	6	2	0.19023
Lyophilization	Time 6	Control Strawberry	6	1	0.304
Lyophilization	Time 6	Control Strawberry	6	2	0.323866
Lyophilization	Time 6	Solarized Strawberry	6	1	0.220927
Lyophilization	Time 6	Solarized Strawberry	6	2	0.234918
Refrigeration	Time 12	Control Strawberry	6	1	1.431708
Refrigeration	Time 12	Control Strawberry	6	2	1.474761
Refrigeration	Time 12	Solarized Strawberry	5	1	1.317231
Refrigeration	Time 12	Solarized Strawberry	6	2	1.365595
Refrigeration	Time 6	Control Strawberry	5	1	1.766688
Refrigeration	Time 6	Control Strawberry	5	2	1.81169
Refrigeration	Time 6	Solarized Strawberry	2	1	1.982583
Refrigeration	Time 6	Solarized Strawberry	2	2	1.994446
Time 0	Time 0	Control Strawberry	6	1	1.70325
Time 0	Time 0	Control Strawberry	6	2	1.718135
Time 0	Time 0	Solarized Strawberry	6	1	1.682781
Time 0	Time 0	Solarized Strawberry	6	2	1.656073

Table S8. Loadings of preservation method-sample combinations on principal components PC1 and PC2 of Figure 3 in the main manuscript.

**Class**	**correlation_with_PC1**	**correlation_with_PC2**
Time 0_Time 0_Control Strawberry	0.642807	-0.79516
Cryopreservation_Time 6_Control Strawberry	0.827818	-0.15492
Refrigeration_Time 6_Control Strawberry	0.72712	-0.12901
Lyophilization_Time 6_Control Strawberry	0.072614	-0.49454
Refrigeration_Time 12_Control Strawberry	0.648225	-0.09797
Cryopreservation_Time 12_Control Strawberry	0.741386	-0.09593
Lyophilization_Time 12_Control Strawberry	0.045251	0.140394
Time 0_Time 0_Solarized Strawberry	0.636676	-0.75341
Cryopreservation_Time 6_Solarized Strawberry	0.788025	-0.20313
Lyophilization_Time 6_Solarized Strawberry	0.127157	-0.10853
Refrigeration_Time 6_Solarized Strawberry	0.759473	-0.145
Refrigeration_Time 12_Solarized Strawberry	0.609742	-0.17932
Cryopreservation_Time 12_Solarized Strawberry	0.625128	-0.02727
Lyophilization_Time 12_Solarized Strawberry	0.091835	-0.07376

Table S9. Loadings of metabolites on principal components PC1 and PC2 of Figure 3 in the main manuscript.

Individual	correlation_with_PC1	correlation_with_PC2
beta-Methyl-D-Glucoside	0.930114	-0.00689
D-Galactonic Acid gamma-Lactone	0.972742	-0.1152
L-Arginine	0.966206	-0.18061
Pyruvic Acid Methyl Ester	0.972632	-0.10784
D-Xylose	0.74063	0.650054
D-Galacturonic Acid	0.966612	-0.14986
L-Asparagine	0.954551	-0.25135
Tween 40	0.947394	-0.25268
i-Erythritol	0.978848	0.102105
2-Hydroxy-Benzoic Acid	0.667868	0.636132
L-Phenylalanine	0.977437	0.010041
Tween 80	0.957998	-0.20754
D-Mannitol	0.927024	-0.29714
4-Hydroxy-Benzoic Acid	0.954654	0.043291
L-Serine	0.97493	-0.09523
alpha-Cyclodextrin	0.87402	-0.22203
N-Acetyl-D-Glucosamine	0.951352	-0.18359
gamma-Hydroxy-Butyric Acid	0.979431	-0.16265
L-Threonine	0.636021	0.522108
Glycogen	0.788712	-0.31619
D-Glucosaminic Acid	0.9759	0.088028
Itaconic Acid	0.960655	0.16854
Glycyl-L-Glutamic Acid	0.961893	0.216427
D-Cellobiose	0.884459	-0.3278
Glucose-1-Phosphate	0.924255	0.275906
alpha-Keto-Butyric Acid	0.728418	0.073203
Phenylethylamine	0.923111	-0.07013
alpha-D-Lactose	0.957687	0.064349
D.L-alpha-Glycerol-Phosphate	0.822787	0.163516
D-Malic Acid	0.951633	0.144621
Putrescine	0.926735	0.252122

Table S10. PERMANOVA and PERMDISP analysis results for fungal communities associated with (solarized) strawberry soil samples. Pseudo-F and F-value indicate the statistic measured by PERMANOVA and PERMDISP, respectively, while the q-value is the false discovery rate (FDR) adjusted p-value. Significance threshold was set at 0.05.

**PERMANOVA**		**PERMDISP**		**Group 1**	**Group 2**
**pseudo-F**	**q-value**	**F-value**	**q-value**		
2.240	0.140	0.130	1.000	Fresh soil	12 months, 4°C storage
2.807	0.136	1.365	0.515	Fresh soil	12 months, -80°C storage
3.381	0.136	3.374	0.289	Fresh soil	12 months, lyophilized storage
1.124	0.316	2.189	0.542	Fresh soil	6 months, 4°C storage
1.271	0.214	0.109	1.000	Fresh soil	6 months, -80°C storage
3.668	0.136	6.611	0.289	Fresh soil	6 months, lyophilized storage
3.567	0.136	0.984	0.542	12 months, 4°C storage	12 months, -80°C storage
3.481	0.136	1.766	0.542	12 months, 4°C storage	12 months, lyophilized storage
4.263	0.136	1.261	0.289	12 months, -80°C storage	12 months, lyophilized storage
2.264	0.136	1.352	0.542	6 months, 4°C storage	12 months, 4°C storage
4.109	0.136	0.294	0.542	6 months, 4°C storage	12 months, -80°C storage
5.934	0.136	0.240	0.515	6 months, 4°C storage	12 months, lyophilized storage
1.492	0.136	0.796	0.754	6 months, 4°C storage	6 months, -80°C storage
8.216	0.136	3.020	0.289	6 months, 4°C storage	6 months, lyophilized storage
2.240	0.138	0.004	1.000	6 months, -80°C storage	12 months, 4°C storage
1.658	0.214	0.593	0.823	6 months, -80°C storage	12 months, -80°C storage
3.225	0.136	1.009	0.542	6 months, -80°C storage	12 months, lyophilized storage
2.933	0.136	1.479	0.289	6 months, -80°C storage	6 months, lyophilized storage
6.216	0.136	2.704	0.289	6 months, lyophilized storage	12 months, 4°C storage
8.423	0.136	7.227	0.289	6 months, lyophilized storage	12 months, -80°C storage
4.417	0.136	1.874	0.289	6 months, lyophilized storage	12 months, lyophilized storage

Table S11. PERMANOVA and PERMDISP analysis results for fungal communities associated with (control) strawberry soil samples. Pseudo-F and F-value indicate the statistic measured by PERMANOVA and PERMDISP, respectively, while the q-value is the false discovery rate (FDR) adjusted p-value. Significance threshold was set at 0.05.

PERMANOVA		PERMDISP		Group 1	Group 2
**pseudo-F**	**q-value**	**F-value**	**q-value**		
2.870	0.140	9.628	0.191	Fresh soil	12 months, 4°C storage
1.860	0.140	24.567	0.191	Fresh soil	12 months, -80°C storage
2.570	0.140	38.209	0.191	Fresh soil	12 months, lyophilized storage
1.280	0.320	29.893	0.191	Fresh soil	6 months, 4°C storage
0.820	0.700	0.000	1.000	Fresh soil	6 months, -80°C storage
2.830	0.140	26.838	0.190	Fresh soil	6 months, lyophilized storage
3.080	0.140	0.400	0.315	12 months, 4°C storage	12 months, -80°C storage
4.220	0.140	2.151	0.191	12 months, 4°C storage	12 months, lyophilized storage
4.260	0.140	1.367	0.191	12 months, -80°C storage	12 months, lyophilized storage
4.390	0.140	0.268	0.191	6 months, 4°C storage	12 months, 4°C storage
4.450	0.140	0.066	0.636	6 months, 4°C storage	12 months, -80°C storage
4.896	0.141	2.835	0.191	6 months, 4°C storage	12 months, lyophilized storage
1.320	0.323	0.909	0.636	6 months, 4°C storage	6 months, -80°C storage
2.853	0.140	0.023	0.750	6 months, 4°C storage	6 months, lyophilized storage
2.930	0.141	0.660	0.681	6 months, -80°C storage	12 months, 4°C storage
1.887	0.141	0.978	0.681	6 months, -80°C storage	12 months, -80°C storage
4.222	0.141	1.435	0.449	6 months, -80°C storage	12 months, lyophilized storage
2.266	0.141	0.871	0.543	6 months, -80°C storage	6 months, lyophilized storage
7.301	0.141	0.184	0.191	6 months, lyophilized storage	12 months, 4°C storage
7.249	0.141	0.133	0.191	6 months, lyophilized storage	12 months, -80°C storage
4.156	0.141	2.995	0.191	6 months, lyophilized storage	12 months, lyophilized storage

Table S12: PERMANOVA and PERMDISP analysis results for bacterial communities associated with (solarized) strawberry soil samples. Pseudo-F and F-value indicate the statistic measured by PERMANOVA and PERMDISP, respectively, while the q-value is the false discovery rate (FDR) adjusted p-value. Significance threshold was set at 0.05.

PERMANOVA		PERMDISP		Group 1	Group 2
pseudo-F	q-value	F-value	q-value		
2.110	0.127	0.082	0.932	Fresh soil	12 months, 4°C storage
2.200	0.130	0.513	0.888	Fresh soil	12 months, -80°C storage
4.943	0.127	0.436	0.252	Fresh soil	12 months, lyophilized storage
2.002	0.127	0.396	0.889	Fresh soil	6 months, 4°C storage
1.266	0.302	0.035	0.889	Fresh soil	6 months, -80°C storage
5.232	0.127	1.190	0.252	Fresh soil	6 months, lyophilized storage
2.775	0.127	0.677	0.314	12 months, 4°C storage	12 months, -80°C storage
6.743	0.127	1.760	0.252	12 months, 4°C storage	12 months, lyophilized storage
6.242	0.127	0.077	0.426	12 months, -80°C storage	12 months, lyophilized storage
2.168	0.127	0.819	0.314	6 months, 4°C storage	12 months, 4°C storage
3.262	0.127	0.067	0.643	6 months, 4°C storage	12 months, -80°C storage
8.836	0.127	<0.001	1.000	6 months, 4°C storage	12 months, lyophilized storage
2.725	0.127	2.207	0.252	6 months, 4°C storage	6 months, -80°C storage
8.081	0.127	0.966	0.252	6 months, 4°C storage	6 months, lyophilized storage
2.768	0.127	0.857	0.314	6 months, -80°C storage	12 months, 4°C storage
3.210	0.127	1.778	0.252	6 months, -80°C storage	12 months, -80°C storage
5.381	0.127	2.973	0.252	6 months, -80°C storage	12 months, lyophilized storage
6.020	0.127	4.687	0.252	6 months, -80°C storage	6 months, lyophilized storage
8.420	0.127	3.370	0.252	6 months, lyophilized storage	12 months, 4°C storage
6.872	0.127	0.207	0.252	6 months, lyophilized storage	12 months, -80°C storage
2.650	0.127	1.366	0.314	6 months, lyophilized storage	12 months, lyophilized storage

Table S13: PERMANOVA and PERMDISP analysis results for bacterial communities associated with (control) strawberry soil samples. Pseudo-F and F-value indicate the statistic measured by PERMANOVA and PERMDISP, respectively, while the q-value is the false discovery rate (FDR) adjusted p-value. Significance threshold was set at 0.05.

**PERMANOVA**		**PERMDISP**		**Group 1**	**Group 2**
**pseudo-F**	**q-value**	**F-value**	**q-value**		
2.480	0.140	10.630	0.230	Fresh soil	12 months, 4°C storage
1.700	0.200	0.880	0.500	Fresh soil	12 months, -80°C storage
3.860	0.140	8.720	0.230	Fresh soil	12 months, lyophilized storage
2.410	0.140	6.640	0.230	Fresh soil	6 months, 4°C storage
1.110	0.390	0.280	0.720	Fresh soil	6 months, -80°C storage
2.820	0.140	0.490	0.230	Fresh soil	6 months, lyophilized storage
2.420	0.140	0.290	0.760	12 months, 4°C storage	12 months, -80°C storage
6.590	0.140	0.180	0.230	12 months, 4°C storage	12 months, lyophilized storage
3.280	0.140	0.210	0.660	12 months, -80°C storage	12 months, lyophilized storage
2.950	0.140	0.430	0.230	6 months, 4°C storage	12 months, 4°C storage
3.380	0.140	0.130	0.960	6 months, 4°C storage	12 months, -80°C storage
7.250	0.140	0.100	0.230	6 months, 4°C storage	12 months, lyophilized storage
2.450	0.140	0.730	0.620	6 months, 4°C storage	6 months, -80°C storage
3.820	0.140	1.990	0.230	6 months, 4°C storage	6 months, lyophilized storage
3.290	0.140	1.140	0.230	6 months, -80°C storage	12 months, 4°C storage
2.160	0.140	0.120	0.700	6 months, -80°C storage	12 months, -80°C storage
4.630	0.140	0.950	0.230	6 months, -80°C storage	12 months, lyophilized storage
3.004	0.139	0.000	1.000	6 months, -80°C storage	6 months, lyophilized storage
4.990	0.140	3.400	0.230	6 months, lyophilized storage	12 months, 4°C storage
3.100	0.140	0.200	0.720	6 months, lyophilized storage	12 months, -80°C storage
2.820	0.140	2.720	0.620	6 months, lyophilized storage	12 months, lyophilized storage

Table S14. Pairwise p-values from the genus-level differential abundance analysis performed using ANCOM-BC2. Rows represent bacterial genera, and columns report the p-values associated with the pairwise contrasts among the experimental groups defined by preservation treatment, storage time, and soil condition.

taxon	p_refrigeration_solarized	p_cryopreservation_solarized	p_lyophilization_solarized	p_refrigeration_control	p_cryopreservation_control	p_lyophilization_control
1959-1	0,199002	0,969031	0,198032	0,322875	0,02505	0,198032
Acidibacter	0,30588	0,755235	1,39E-09	0,542353	0,928614	1,39E-09
Acidicaldus	0,616259	0,69517	0,250752	0,559114	0,010607	0,250752
Acidiferrimicrobium	0,302976	0,105867	6,05E-08	0,173883	0,915359	6,05E-08
Acidothermus	0,87531	0,98104	0,806798	0,997306	0,96353	0,806798
Actinocorallia	0,076486	0,753106	0,683722	0,003199	0,001345	0,683722
Actinomadura	0,107395	0,840748	0,154874	0,903646	0,313048	0,154874
Agromyces	0,027063	1,91E-05	8,65E-16	0,240132	0,02221	8,65E-16
Alkalicoccobacillus	0,926859	0,957209	0,971709	0,965541	0,798819	0,971709
Allomeiothermus	0,00581	0,019418	3,4E-05	0,586622	0,010512	3,4E-05
Alsobacter	0,953392	0,996041	0,962838	0,971992	0,926504	0,962838
Amaricoccus	0,820754	0,831908	0,682151	0,875917	0,823556	0,682151
Ammoniibacillus	0,13614	0,116669	0,986069	0,31552	9,79E-06	0,986069
Ammoniphilus	0,708778	0,95266	0,947944	0,642283	0,868821	0,947944
Anaerobacillus	0,998667	0,916158	0,955496	0,958708	0,912031	0,955496
Anaerolinea	9,94E-06	0,146891	7,42E-05	0,523348	0,857894	7,42E-05
Anaeromyxobacter	0,057124	7,1E-06	0,058667	0,113113	0,000166	0,058667
Aneurinibacillus	0,830913	0,01478	0,17831	0,463288	0,521964	0,17831
Anoxybacillus	0,001528	0,000222	0,00015	0,976232	6,31E-05	0,00015
Archangium	0,736007	0,292019	0,321234	0,503928	0,274203	0,321234
Arenimonas	0,300481	0,180815	0,025989	0,161969	0,722754	0,025989
Arthrobacter	0,078356	0,786221	1,7E-15	0,51945	0,55071	1,7E-15
Azospirillum	0,893427	0,7085	0,860157	0,858741	0,77152	0,860157
BBMC-4	0,732299	0,924498	0,667083	0,99825	0,705773	0,667083
Bacillus	0,6052	0,346449	0,938416	0,131374	0,37556	0,938416
Baekduia	0,806661	0,382206	0,127682	0,918562	0,000107	0,127682
Blastococcus	0,943723	0,488178	0,364252	0,201443	0,277781	0,364252
Bradyrhizobium	0,946691	0,993913	0,263575	0,827178	0,90121	0,263575
Brevibacillus	0,00133	0,2245	0,004618	1,94E-05	0,320435	0,004618
Brucella	0,343035	0,102431	0,97692	0,289794	0,840619	0,97692
Bryobacter	0,025402	0,000387	4,01E-05	0,000384	0,031709	4,01E-05
Burkholderia-Caballeronia-Paraburkholderia	0,240804	0,422704	5,68E-07	0,454833	0,774232	5,68E-07
Caldalkalibacillus	0,242753	0,000154	0,053315	0,798648	8,1E-08	0,053315
Caldicoprobacter	0,638217	0,807073	0,69986	0,557855	0,22237	0,69986
Caldimonas	0,784437	0,626908	0,740082	0,742558	0,870744	0,740082
Calidithermus	0,910478	0,933563	0,72256	0,967979	0,960056	0,72256
Candidatus Alysiosphaera	0,971361	0,914878	0,796542	0,838092	0,83332	0,796542
Candidatus Koribacter	0,844564	0,040933	0,000338	0,000304	0,07599	0,000338
Candidatus Nitrosocosmicus	0,007674	0,035061	0,260833	0,71275	0,000195	0,260833
Candidatus Solibacter	0,094427	0,022709	0,000968	0,115426	0,00443	0,000968

Candidatus Udaeobacter	0,091473	0,63785	4,73E-08	0,061081	0,113684	4,73E-08
Candidatus Xiphinematobacter	0,896692	0,96581	0,708242	0,997444	0,953942	0,708242
Cavicella	0,729054	0,820029	0,695368	0,731785	0,80478	0,695368
Cellulosimicrobium	0,207589	0,000985	0,180167	0,446762	0,243873	0,180167
Chthoniobacter	0,033268	0,402643	0,786713	0,593493	0,344541	0,786713
Clostridium	0,083379	0,568148	0,217278	0,030612	0,076079	0,217278
Cohnella	0,746359	0,989035	0,931552	0,928529	0,565125	0,931552
Compostibacillus	0,210853	0,470205	0,988234	0,582766	0,062714	0,988234
Corallococcus	0,366889	0,003712	0,112662	0,000777	0,405474	0,112662
Croceibacterium	0,858247	0,050228	3,31E-05	0,982314	0,012241	3,31E-05
Dankookia	0,948712	0,926292	0,856205	0,976173	0,880577	0,856205
Desertimonas	0,993428	0,849835	0,827729	0,933036	0,995978	0,827729
Devosia	0,000322	0,220091	2,29E-07	0,000442	0,120173	2,29E-07
Dokdonella	0,650831	0,930683	0,125069	0,712839	0,93898	0,125069
Dongia	0,470698	0,02387	1,55E-06	0,339285	0,020766	1,55E-06
Effusibacillus	0,022071	0,15574	0,793361	0,121331	0,049601	0,793361
Ellin6067	0,032802	0,970725	1,46E-09	0,001183	0,392424	1,46E-09
Ensifer	1,94E-06	0,000209	2,6E-10	0,01729	0,112408	2,6E-10
FCPS473	0,231423	0,086313	0,229879	0,145624	0,372779	0,229879
Fictibacillus	0,696768	0,725041	0,288259	0,843179	0,599833	0,288259
Flavisolibacter	0,015983	0,33416	0,00023	0,000183	0,660424	0,00023
Flavitalea	0,882237	0,869024	0,369544	0,910508	0,994426	0,369544
Flavobacterium	0,000275	4,72E-09	5,71E-17	0,765543	0,035462	5,71E-17
Gaiella	0,078893	0,592046	0,720106	0,00232	0,915344	0,720106
Geminicoccus	0,895827	0,896716	0,881279	0,845519	0,806246	0,881279
Gemmata	3,8E-06	0,189884	3,05E-07	0,002315	0,278393	3,05E-07
Gemmatimonas	1,64E-08	0,747958	4,5E-06	2,79E-10	0,593095	4,5E-06
Gracilibacter	0,082694	0,258391	0,019368	0,929071	0,43448	0,019368
HN-HF0106	0,026847	0,412485	0,713406	0,0077	0,020869	0,713406
Halocella	0,045031	0,751809	0,147796	0,653712	0,01578	0,147796
Haloplasma	0,887607	0,985788	0,715876	0,842432	0,656204	0,715876
Herbinix	0,505002	0,419903	0,96836	0,406663	0,000755	0,96836
Hydrogenispora	0,052798	0,007253	0,885198	0,249354	0,001222	0,885198
Hyphomicrobium	0,556705	0,967065	5,25E-16	0,72736	0,00175	5,25E-16
Ilumatobacter	0,873952	0,895302	0,747907	0,985789	0,961386	0,747907
Jatrophihabitans	0,981526	0,976943	0,846714	0,933389	0,932723	0,846714
Kitasatospora	0,925631	0,967271	0,745256	0,844062	0,760764	0,745256
Knoellia	0,023199	0,068093	1,67E-07	0,00948	0,000793	1,67E-07
Kribbella	0,248663	0,692868	0,004788	0,003242	0,245367	0,004788
Kroppenstedtia	0,067309	0,081026	0,001986	0,548019	0,000472	0,001986
Legionella	0,959408	0,624702	0,559754	0,714704	0,57418	0,559754
Leifsonia	0,784918	0,837826	0,70111	0,938847	0,897904	0,70111
Limnochorda	0,944042	0,03631	0,223783	0,670232	0,000217	0,223783
Litorilinea	0,416536	0,054369	0,734142	0,893022	0,044792	0,734142
Longispora	0,000666	7,44E-07	5,96E-07	0,00823	1,21E-10	5,96E-07
Luteimonas	0,896465	0,912114	0,702614	0,864855	0,924014	0,702614
Luteitalea	0,953504	0,830924	0,477279	0,878609	0,957464	0,477279

Lutispora	0,487587	0,574191	0,035359	0,511869	0,498838	0,035359
Lysinibacillus	0,907308	0,000454	0,020313	0,009705	0,162706	0,020313
Lysobacter	0,031476	0,003331	4,26E-17	5,29E-06	0,054068	4,26E-17
MND1	1,1E-06	0,803648	7,57E-06	1,89E-08	0,985054	7,57E-06
Marmoricola	0,75103	0,073093	0,255773	0,520688	0,508522	0,255773
Massilia	0,222278	0,074874	0,66591	0,593463	0,072877	0,66591
Mesobacillus	0,796454	0,881097	0,999372	0,959978	0,819445	0,999372
Methanoculleus	0,242669	0,786266	0,746635	0,431575	4,4E-05	0,746635
Methanosarcina	0,850549	0,053145	1,09E-07	0,193784	1,63E-08	1,09E-07
Microbispora	0,000137	0,001042	3,13E-06	0,509437	0,24855	3,13E-06
Microvirga	0,986895	0,983595	6,47E-06	0,249577	0,107086	6,47E-06
Nannocystis	0,965964	0,92496	0,872299	0,858695	0,875243	0,872299
Neobacillus	0,164006	0,029714	1,3E-05	0,42159	0,050889	1,3E-05
Niallia	0,829447	0,00413	9,03E-05	0,903632	0,710513	9,03E-05
Niastella	0,980742	0,832732	0,416191	0,825372	0,972153	0,416191
Nitrolancea	0,014064	0,510748	0,000289	0,802711	0,926969	0,000289
Nitrosospira	0,387846	0,723289	4,52E-05	0,202503	0,736866	4,52E-05
Nitrospira	0,333491	0,122128	3,66E-12	0,200349	0,17896	3,66E-12
Nocardia	0,984202	0,908073	0,778539	0,950895	0,950397	0,778539
Nocardioides	0,724396	0,768202	0,839189	0,284103	0,731883	0,839189
Nonomuraea	0,598553	0,761381	0,613187	0,845039	0,001149	0,613187
Nordella	0,94957	0,867756	0,000334	0,499291	0,286247	0,000334
Novibacillus	0,527298	0,235127	0,60481	0,570181	0,027439	0,60481
Noviherbaspirillum	6,96E-05	2,44E-07	0,785315	0,083976	0,691728	0,785315
Novosphingobium	0,393921	0,616597	0,229065	0,997194	0,833699	0,229065
OM27 clade	0,383373	0,107756	0,001134	0,92602	0,085744	0,001134
Oceanobacillus	0,906311	0,957674	0,958154	0,89091	0,756632	0,958154
Oligoflexus	0,50305	0,998895	0,07666	0,886242	0,261375	0,07666
Opitutus	0,464383	0,132439	0,008484	0,116785	0,606271	0,008484
Oryzihumus	0,185502	0,445751	0,88704	0,726713	0,20734	0,88704
Paenarthrobacter	0,103417	0,844606	0,00027	0,005966	0,000654	0,00027
Paenibacillus	0,386122	0,87909	0,750112	0,895211	0,681057	0,750112
Paeniclostridium	0,470107	0,083129	5,29E-05	0,74239	0,0092	5,29E-05
Paenisporosarcina	0,903747	0,982896	0,952787	0,971531	0,727429	0,952787
Paraclostridium	0,919229	0,907333	0,95255	0,959098	0,897234	0,95255
Paraconexibacter	0,930872	0,787764	0,867724	0,878106	0,82711	0,867724
Parviterribacter	0,831932	0,32062	0,099908	0,092389	0,866528	0,099908
Pedococcus-Phycicoccus	0,543024	0,13456	2,41E-05	0,766177	0,193889	2,41E-05
Pedomicrobium	0,845799	0,093784	1,04E-10	0,937055	0,000363	1,04E-10
Peribacillus	0,299574	0,036654	1,31E-05	0,009992	0,567386	1,31E-05
Phenylobacterium	0,008262	0,121126	0,208438	0,200813	0,303096	0,208438
Pir4 lineage	0,78761	0,937822	0,468135	0,828386	0,75595	0,468135
Pirellula	0,039762	0,572683	8,88E-11	0,195593	0,767332	8,88E-11
Planifilum	0,054914	0,005432	0,012596	0,216665	0,000165	0,012596
Priestia	0,045228	0,000452	9,32E-06	0,474709	0,081468	9,32E-06
Proteiniborus	0,024963	0,153289	0,169351	0,994404	0,392415	0,169351
Pseudaminobacter	0,089844	0,194759	1,22E-05	0,184273	0,640707	1,22E-05
Pseudomonas	0,20971	0,384807	0,067188	0,943997	0,937545	0,067188

Psychrobacillus	0,218015	0,9752	0,20945	0,236547	0,000487	0,20945
Puia	0,874184	0,738068	0,918573	0,974988	0,959124	0,918573
Ramlibacter	0,885225	0,91479	0,285531	0,706371	0,854186	0,285531
Reyranella	0,411974	0,782	0,920076	0,317248	0,628724	0,920076
Rhizobium	0,67207	0,807802	0,207991	0,97807	0,905599	0,207991
Rhodopila	0,870678	0,893058	0,986498	0,981409	0,870291	0,986498
Romboutsia	0,986335	0,148431	0,000931	0,333935	0,002291	0,000931
Roseicella	0,949897	0,24754	0,347137	0,250875	0,159805	0,347137
Roseomonas	0,435332	0,188477	0,389622	0,575685	0,65818	0,389622
Rummeliibacillus	0,867826	0,970294	0,962863	0,98104	0,958182	0,962863
SH-PL14	0,886884	0,886037	0,521091	0,893969	0,842294	0,521091
Saccharomonospora	0,775435	0,302195	0,849737	0,859829	0,348634	0,849737
Shouchella	0,938294	0,937768	0,729635	0,902976	0,762772	0,729635
Sinibacillus	0,185269	0,676179	0,022633	0,984409	0,007645	0,022633
Skermanella	0,765472	0,66307	0,000152	0,008534	0,45857	0,000152
Solibacillus	0,819858	0,224021	0,001243	0,262751	0,265723	0,001243
Solirubrobacter	0,559808	0,101129	0,027036	0,907303	9,71E-08	0,027036
Sphaerisporangium	0,008433	0,734184	0,155556	0,080903	0,87947	0,155556
Sphaerobacter	0,87393	0,743291	0,580324	0,777957	0,628715	0,580324
Sphingomonas	0,000276	0,976457	5,08E-08	6,57E-06	0,036196	5,08E-08
Sporomusa	0,934777	0,942316	0,991313	0,9507	0,884443	0,991313
Sporosarcina	0,960755	0,976248	0,982292	0,987151	0,817697	0,982292
Stenotrophobacter	0,331762	0,39646	0,658953	0,31754	0,397143	0,658953
Steroidobacter	0,97528	0,048824	4,08E-12	0,269097	0,424681	4,08E-12
Streptomyces	0,641637	0,037736	0,00476	0,22012	1,12E-06	0,00476
Streptosporangium	0,995893	0,262231	0,7895	0,548154	0,057876	0,7895
Sumerlaea	0,051787	0,852416	1,14E-06	0,002338	0,112813	1,14E-06
Symbiobacterium	0,004974	0,001644	0,922632	0,874559	5,23E-05	0,922632
Syntrophaceticus	0,005388	0,004736	7,87E-05	0,154539	0,000435	7,87E-05
Tepidanaerobacter	0,874134	0,900987	0,956269	0,702678	0,940285	0,956269
Tepidimicrobium	0,149989	0,479696	0,057595	0,186859	0,056353	0,057595
Terrabacter	0,302585	0,68109	0,002066	0,235637	0,509238	0,002066
Terrimonas	0,696397	0,818837	0,587492	0,776511	0,929711	0,587492
Thalassiella	0,389047	0,007404	0,028704	0,199871	0,008523	0,028704
Thermincola	0,002386	0,270136	0,474527	0,016075	0,162121	0,474527
Thermobacillus	0,024269	0,039248	0,179194	0,326676	6,19E-06	0,179194
Thermocatellispora	0,004057	2,72E-07	0,001407	0,887594	0,001316	0,001407
Thermoflavimicrobium	0,398669	0,965937	0,536649	0,719449	0,781145	0,536649
Thermomonospora	0,911581	0,001724	0,356873	0,279417	0,002202	0,356873
Thermopolyspora	7,8E-05	0,079265	0,057131	0,144771	7,87E-06	0,057131
Thermosporothrix	0,860583	0,904109	0,870434	0,827714	0,931099	0,870434
Tumebacillus	0,674494	0,45448	0,00249	0,663671	0,03364	0,00249
Turicibacter	0,683318	0,134607	0,000827	0,625657	9,94E-07	0,000827
UCG-012	0,35818	0,789827	0,314378	0,171265	0,00098	0,314378
Unclassified_67-14	3,33E-05	0,006785	0,104188	0,117128	0,000288	0,104188
Unclassified_AKIW781	0,123828	0,614036	4,99E-09	0,054731	0,059421	4,99E-09
Unclassified_AKYG1722	0,000158	0,23398	0,37549	0,004668	0,069315	0,37549

Unclassified_Acetobacteraceae	1,57E-07	0,006758	3,54E-12	0,034772	0,418988	3,54E-12
Unclassified_Acidimicrobiaceae	0,003665	0,550356	0,473984	0,011179	0,0144	0,473984
Unclassified_Anaerolineaceae	0,809684	0,018428	0,006554	0,151694	0,826526	0,006554
Unclassified_BIrii41	0,972665	0,878695	0,000128	0,004252	0,662418	0,000128
Unclassified_Bacillaceae	0,056789	0,108051	0,32891	0,010569	0,000127	0,32891
Unclassified_Blastocatellaceae	0,329252	0,423107	0,97712	0,361779	0,428749	0,97712
Unclassified_Caldilineaceae	0,533398	0,666328	9,95E-08	0,02343	0,324971	9,95E-08
Unclassified_Chitinophagaceae	0,87138	0,778861	0,636353	0,866584	0,758211	0,636353
Unclassified_Comamonadaceae	0,616038	0,825746	0,251612	0,528592	0,949404	0,251612
Unclassified_Fimbriimonadaceae	2,78E-06	0,009072	2,54E-05	0,01187	0,434384	2,54E-05
Unclassified_Geminicoccaceae	0,881074	0,891797	0,752867	0,853157	0,751461	0,752867
Unclassified_Gemmataceae	0,370213	0,000407	0,10446	0,143073	0,164227	0,10446
Unclassified_Gemmatimonadaceae	2,1E-06	0,599611	3E-11	5,56E-07	0,777421	3E-11
Unclassified_Haliangiaceae	0,730356	0,742536	0,989672	0,626043	0,734489	0,989672
Unclassified_Heliobacteriaceae	0,98898	0,96585	0,820645	0,882503	0,841342	0,820645
Unclassified_Hyphomicrobiaceae	0,020916	0,018924	1,1E-05	0,378031	0,01795	1,1E-05
Unclassified_Ilumatobacteraceae	0,783646	0,175695	1,29E-13	0,991055	9,83E-05	1,29E-13
Unclassified_Intrasporangiaceae	0,95493	0,178093	3,99E-07	0,68088	0,915307	3,99E-07
Unclassified_JG30-KF-AS9	0,533507	0,153418	0,276419	0,595149	0,737986	0,276419
Unclassified_JG30-KF-CM45	0,05517	0,896148	0,589788	0,052175	0,00373	0,589788
Unclassified_Ktedonobacteraceae	0,356628	0,14971	0,140393	0,758704	0,065491	0,140393
Unclassified_LWQ8	0,003804	0,874621	7,3E-08	0,002821	0,777275	7,3E-08
Unclassified_Lachnospiraceae	0,2812	0,121204	0,145434	0,935948	0,818742	0,145434
Unclassified_Limnochordaceae	0,004929	0,006767	0,084319	0,032948	0,001856	0,084319
Unclassified_Longimicrobiaceae	0,915676	0,937022	0,849252	0,932875	0,731454	0,849252
Unclassified_Marinococcaceae	0,89959	0,608325	0,581089	0,978675	0,921583	0,581089
Unclassified_Methyloligellaceae	0,898576	0,759352	2,61E-21	0,755421	0,041164	2,61E-21
Unclassified_Micrococcaceae	0,011821	0,743909	2,03E-15	0,343233	0,738898	2,03E-15
Unclassified_Micropepsaceae	0,416279	0,399005	0,628536	0,376419	0,372006	0,628536

Unclassified_Microscillaceae	1,28E-12	0,169296	0,257066	3,13E-13	0,174057	0,257066
Unclassified_Moraxellaceae	0,244363	0,788049	0,077542	0,1484	0,585929	0,077542
Unclassified_Myxococcaceae	0,828539	0,963136	0,640067	0,853887	0,991655	0,640067
Unclassified_Nitrososphaeraceae	0,987054	0,88729	0,655525	0,94841	0,922427	0,655525
Unclassified_Paenibacillaceae	0,984523	0,539669	0,021325	0,666313	0,003561	0,021325
Unclassified_Pedosphaeraceae	0,000755	0,138683	0,673512	1,62E-12	0,001223	0,673512
Unclassified_Peptococcaceae	0,53319	0,12619	0,07429	0,776307	3,8E-05	0,07429
Unclassified_Pirellulaceae	0,789497	0,69743	0,471307	0,797995	0,947485	0,471307
Unclassified_Roseiflexaceae	0,004311	0,060861	0,004629	0,127852	0,746274	0,004629
Unclassified_SC-I-84	3,37E-05	0,304892	1,37E-10	2,86E-09	0,145613	1,37E-10
Unclassified_Sandaracinaceae	0,044212	0,365698	1,91E-05	0,400975	0,000458	1,91E-05
Unclassified_Solirubrobacteraceae	0,000988	0,274841	0,482157	0,036418	0,129996	0,482157
Unclassified_Sporolactobacillaceae	0,42281	0,151985	0,177456	0,359744	0,341866	0,177456
Unclassified_Symbiobacteraceae	0,68712	0,42798	0,069769	0,300062	0,945379	0,069769
Unclassified_TRA3-20	0,384349	0,967354	0,23339	0,349839	0,891181	0,23339
Unclassified_Thermoactinomycetaceae	0,411276	0,345685	0,949915	0,180951	0,014782	0,949915
Unclassified_Vicinamibacteraceae	0,087721	0,119273	1,03E-09	3,91E-05	0,44065	1,03E-09
Unclassified_WD2101 soil group	0,313955	0,236429	1,78E-08	0,027898	0,434974	1,78E-08
Unclassified_Xanthobacteraceae	0,684543	0,604466	0,592721	0,563362	0,926419	0,592721
Unclassified_YM_S32_TM7_50_20	0,750545	0,801872	0,537112	0,640937	0,983857	0,537112
Unclassified_env.OPS 17	0,001909	0,070923	0,659436	0,000397	0,220335	0,659436
Unclassified_unknown	0,001076	0,978349	1,87E-07	0,000163	0,864117	1,87E-07
Ureibacillus	0,997633	0,626345	0,4658	0,495358	0,137706	0,4658
Vulgatibacter	0,90434	0,891345	0,931872	0,865617	0,92243	0,931872
Zavarzinella	0,770519	0,873249	0,951411	0,969613	0,984644	0,951411
mle1-7	0,276609	0,329005	1,39E-10	0,002088	0,198927	1,39E-10

Table S15. Pairwise p-values from the genus-level differential abundance analysis performed using ANCOM-BC2. Rows represent fungal genera, and columns report the p-values associated with the pairwise contrasts among the experimental groups defined by preservation treatment, storage time, and soil condition.

taxon	p_refrigeration_solarized	p_cryopreservation_solarized	p_lyophilization_solarized	p_refrigeration_control	p_cryopreservation_control	p_lyophilization_control
Aaosphaeria	0,69271	0,552814	0,264796	0,837009	0,556723	0,264796
Acaulium	0,87512	0,975387	0,91413	0,870326	0,970956	0,91413
Acremonium	0,020592	0,02869	0,080679	0,743188	0,387794	0,080679
Acrophialophora	0,723513	0,289301	0,435261	0,348869	0,887537	0,435261
Actinomortierella	2,81E-05	0,001948	0,000591	0,008958	0,884658	0,000591
Actinomucor	0,562648	0,574868	0,209938	0,628995	0,421923	0,209938
Alternaria	1,7E-06	0,095499	0,087605	0,002818	0,77916	0,087605
Aphelidiomycota_gen_Incertae_sedis	0,663805	0,660077	0,772182	0,984119	0,908523	0,772182
Ascobolus	0,002274	0,194704	0,248718	0,170838	0,034368	0,248718
Ascospirella	0,811995	0,927022	0,685278	0,989606	0,877377	0,685278
Aspergillus	0,002708	1,56E-07	0,00298	0,098626	0,139048	0,00298
Basidiobolales_gen_Incertae_sedis	0,80283	0,982107	0,958563	0,715654	0,983056	0,958563
Basidiobolomycota_gen_Incertae_sedis	0,884255	0,855595	0,874243	0,881219	0,623862	0,874243
Capturomyces	0,859397	0,853118	0,941031	0,754132	0,707094	0,941031
Cephalotrichum	0,460818	0,493754	0,739443	0,138788	0,92561	0,739443
Chaetomium	0,202906	0,598222	0,379503	0,483247	0,875857	0,379503
Chloridium	0,87553	0,744137	0,910914	0,77142	0,85731	0,910914
Chordomyces	0,757074	0,757385	0,744337	0,901081	0,874588	0,744337
Chrysosporium	0,991536	0,87347	0,965945	0,904536	0,725747	0,965945
Chytridiomycota_gen_Incertae_sedis	0,74474	0,909236	0,702133	0,656758	0,894361	0,702133
Cladorrhinum	1,52E-08	0,653948	0,000626	6,02E-07	6,96E-05	0,000626
Cladosporium	1,25E-08	3,5E-05	6,09E-06	2,4E-06	0,533166	6,09E-06
Clonostachys	0,000423	0,17204	0,001992	0,059994	0,403018	0,001992
Coniochaeta	0,502306	0,938716	0,79172	0,883412	0,756187	0,79172
Conlarium	0,812561	0,975266	0,86684	0,896693	0,844946	0,86684
Coprinopsis	0,012862	0,299828	0,529958	0,432695	0,314387	0,529958
Corynascella	0,972219	0,998323	0,773989	0,948724	0,831781	0,773989
Cryptococcus	0,92951	0,714601	0,868861	0,917369	0,84078	0,868861
Cunninghamella	0,926637	0,963169	0,808884	0,980677	0,626831	0,808884
Curvularia	0,202675	0,300294	0,275898	0,054041	0,974274	0,275898
Cystofilobasidium	0,767583	0,984488	0,927631	0,873927	0,708525	0,927631
Debaryomyces	0,985213	0,822663	0,779007	0,960459	0,822005	0,779007
Dendryphion	0,888127	0,98405	0,815447	0,813448	0,972366	0,815447
Dimorphiseta	0,501108	0,481474	0,018564	0,569057	0,858297	0,018564
Emmonsiellopsis	0,752208	0,945248	0,855533	0,649885	0,895898	0,855533
Endogonomycetes_gen_Incertae_sedis	0,430005	0,910307	0,728246	0,764993	0,462721	0,728246
Enterocarpus	0,689984	0,020461	0,320337	0,211931	0,859944	0,320337
Exophiala	0,878378	0,936314	0,880469	0,711947	0,948484	0,880469
Extremopsis	0,625678	0,627099	0,968952	0,572751	0,756304	0,968952

Fungi_gen_Incertae_se dis	0,578472	0,932429	0,094261	0,363913	0,809114	0,094261
Fusarium	0,000827	0,208521	0,002817	0,987608	0,159343	0,002817
GS11_gen_Incertae_se dis	0,000967	0,018654	0,199514	0,562792	0,844783	0,199514
Geomyces	0,779505	0,709108	0,692729	0,882027	0,85117	0,692729
Gibellulopsis	0,282895	0,916393	0,016345	0,328675	0,584068	0,016345
Humicola	0,007077	0,000963	8,59E-07	0,155599	0,109208	8,59E-07
Hyalorbilia	0,973162	0,884133	0,679379	0,740913	0,874256	0,679379
Hyaloscyphaceae_gen _Incertae_sedis	0,899479	0,965889	0,702335	0,360696	0,567722	0,702335
Hypocreales_gen_Ince rtae_sedis	0,20013	0,597171	0,213279	0,807253	0,537582	0,213279
Kernia	0,643095	0,123012	0,810904	0,238515	0,803702	0,810904
Leptoxyphium	0,116566	0,265465	0,000813	3,46E-05	0,003213	0,000813
Leucothecium	0,943694	0,842487	0,945543	0,870377	0,879107	0,945543
Linnemannia	0,326944	0,016013	0,000191	0,141366	0,990941	0,000191
Lipomyces	0,285849	0,152463	0,944053	0,546854	0,102959	0,944053
Lobulomycetales_gen_ Incertae_sedis	0,694782	0,52501	0,77857	0,608566	0,925813	0,77857
Macrophomina	0,525784	0,889827	0,795307	0,874639	0,694979	0,795307
Marquandomyces	0,81645	0,368232	0,444865	0,691724	0,679799	0,444865
Melanocarpus	0,003603	0,791943	0,094057	0,22357	0,112762	0,094057
Metacordyceps	0,778542	0,985414	0,862836	0,817337	0,982817	0,862836
Metarhizium	0,717657	0,001873	0,00067	0,541085	0,047672	0,00067
Microascaceae_gen_In certae_sedis	0,823186	0,001454	0,726754	0,027083	0,103613	0,726754
Microdochium	0,868933	0,886912	0,993902	0,75924	0,820456	0,993902
Mortierella	0,835443	0,174746	0,002755	0,004943	0,114008	0,002755
Mucor	0,586847	0,000777	0,002846	0,213045	0,700521	0,002846
Mycothermus	0,070102	0,351349	0,236076	0,315097	0,190984	0,236076
Neopestalotiopsis	0,585694	0,3832	0,883844	0,864867	0,861946	0,883844
Niesslia	0,714847	0,860818	0,679208	0,531711	0,788727	0,679208
Ochroconis	0,937933	0,961325	0,948722	0,966991	0,864337	0,948722
Olpidium	0,413929	0,12495	0,955512	0,002584	0,776039	0,955512
Papiliotrema	0,00759	0,22688	0,496411	0,008317	0,609037	0,496411
Paraphaeosphaeria	0,312786	0,587848	0,157124	0,507142	0,307745	0,157124
Penicillium	0,026136	0,807188	0,584577	0,804765	0,086036	0,584577
Pezizomycotina_gen_I ncertae_sedis	0,238753	0,54502	0,58314	0,676227	0,960368	0,58314
Phialophora	0,495941	0,387428	0,355982	0,696039	0,74634	0,355982
Pilidium	0,220845	0,021792	0,22456	0,619827	0,974009	0,22456
Plectosphaerella	0,597494	0,446262	0,08391	0,980458	0,803448	0,08391
Podospora	0,002649	0,831831	0,352659	0,245276	6,41E-06	0,352659
Preussia	0,056855	0,050106	0,289655	0,747928	0,595305	0,289655
Psathyrella	0,397194	0,718081	0,93679	0,132727	0,612457	0,93679
Pseudeurotium	0,256341	0,619479	0,235919	0,89626	0,908635	0,235919
Pseudocyclothyriella	0,969901	0,786846	0,733516	0,939773	0,848878	0,733516
Pseudogymnoascus	0,428056	0,908646	0,966477	0,809157	0,922988	0,966477
Pseudopyrenochaeta	0,744199	0,576139	0,9868	0,624248	0,639166	0,9868

Pseudorhypophila	0,007429	0,948881	0,192526	0,513483	0,011659	0,192526
Pseudothielavia	8,9E-05	0,000749	0,009478	0,411922	0,812194	0,009478
Pulvinulaceae_gen_Incertae_sedis	0,967878	0,643563	0,396156	0,989298	0,829729	0,396156
Purpureocillium	0,007154	0,420868	0,265856	0,33697	0,147587	0,265856
Rhizophydiaceae_gen_Incertae_sedis	0,918624	0,875169	0,74927	0,969776	0,96595	0,74927
Rhizopus	0,550904	0,709464	0,005692	0,407706	0,781156	0,005692
Rhodotorula	0,376576	0,618333	0,296699	0,560031	0,851153	0,296699
Rozellomycota_gen_Incertae_sedis	0,754552	0,766109	0,601275	0,881735	0,682062	0,601275
Sagenomella	0,431463	0,401713	0,95076	0,620533	0,78835	0,95076
Saitozyma	0,715751	0,50865	0,013444	0,963922	0,008769	0,013444
Sanchytrium	0,910868	0,926341	0,609185	0,983179	0,650749	0,609185
Schizothecium	0,215232	0,315599	0,062347	0,598543	0,486347	0,062347
Sclerostagonospora	0,630821	0,620075	0,892437	0,986397	0,527264	0,892437
Scolecobasidium	0,004132	0,140331	0,030493	0,605108	0,054029	0,030493
Scopulariopsis	0,904866	0,858727	0,767063	0,900353	0,885706	0,767063
Scutellinia	0,880351	0,620766	0,726173	0,897897	0,777277	0,726173
Scytalidium	3,62E-05	5,14E-06	0,000352	0,997211	0,803957	0,000352
Setophoma	0,73559	0,30192	0,313986	0,581225	0,058575	0,313986
Sodiomyces	0,962241	0,961785	0,674494	0,883251	0,710826	0,674494
Solicoccozyma	0,520504	0,648906	0,002506	0,439462	0,020188	0,002506
Spiromastigoides	0,831765	0,635111	0,310546	0,751428	0,488955	0,310546
Spizellomyces	0,929239	0,794931	0,563204	0,62563	0,864205	0,563204
Spizellomycetaceae_gen_Incertae_sedis	0,926495	0,71243	0,869204	0,417556	0,553791	0,869204
Spizellomycetales_gen_Incertae_sedis	0,161566	0,443935	0,986897	0,762714	0,476381	0,986897
Stachybotrys	0,018958	2,7E-07	1,87E-05	0,163194	0,004282	1,87E-05
Stephanonectria	0,829193	0,787455	0,913621	0,840935	0,982175	0,913621
Striatibotrys	0,973252	0,875261	0,973093	0,914684	0,86225	0,973093
Striaticonidium	0,952036	0,807781	0,778035	0,881889	0,99712	0,778035
Syncephalis	0,666468	0,237974	0,166786	0,066225	0,110617	0,166786
Talaromyces	0,040816	0,309968	0,037977	0,170312	0,383952	0,037977
Tengochaeta	0,096486	0,00025	0,083256	0,136587	0,086433	0,083256
Tetracladium	0,852343	0,743988	0,89372	0,843615	0,348831	0,89372
Thelonectria	0,011715	0,023205	0,787597	0,543057	0,119175	0,787597
Thermomyces	0,045819	0,440637	0,056818	0,506851	0,717985	0,056818
Triangularia	0,935387	0,429983	0,10008	0,810298	0,242599	0,10008
Trichoderma	4,62E-10	2,46E-06	2,71E-06	0,819551	0,162963	2,71E-06
Trichophyton	0,889652	0,970048	0,933701	0,89852	0,929569	0,933701
Trichosporiella	0,77347	0,808477	0,569759	0,928642	0,97412	0,569759
Triscelophorus	0,836234	0,939031	0,703695	0,859117	0,988588	0,703695
Unclassified_Chaetomiaceae	0,93797	0,114592	0,315673	0,550012	0,729201	0,315673
Unclassified_Coniochaetaceae	0,832978	0,241085	0,239314	0,495993	0,494161	0,239314
Unclassified_Didymellaceae	0,005835	8,9E-07	0,000958	0,584299	0,327805	0,000958

Unclassified_Mortierellaceae	0,020772	0,153128	0,041	0,008991	0,898105	0,041
Unclassified_Spiromastigaceae	1	1	1	1	1	1
Unclassified_Stictidaceae	0,783167	0,882456	0,736819	0,85754	0,91951	0,736819
Unclassified_unknown	3,3E-05	0,77325	0,966429	0,682963	0,952516	0,966429
Vishniacozyma	6,48E-07	0,009769	1,14E-05	8,42E-07	0,560367	1,14E-05
Waltergamsia	0,987248	0,943088	0,818763	0,850081	0,97447	0,818763

SUPPLEMENTARY FIGURES

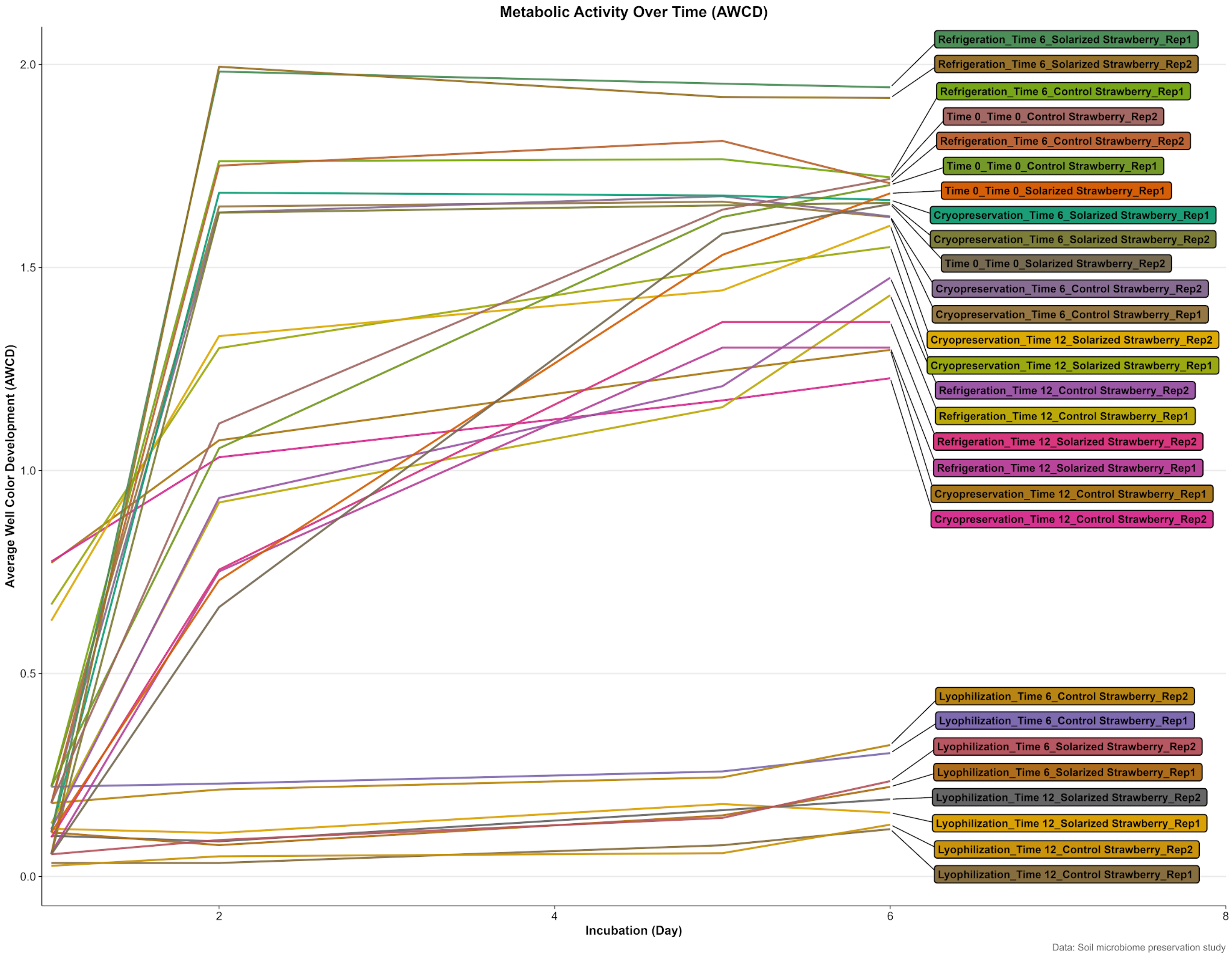

Figure S1. AWCD curves of BIOLOG EcoPlates of soil samples during incubation.

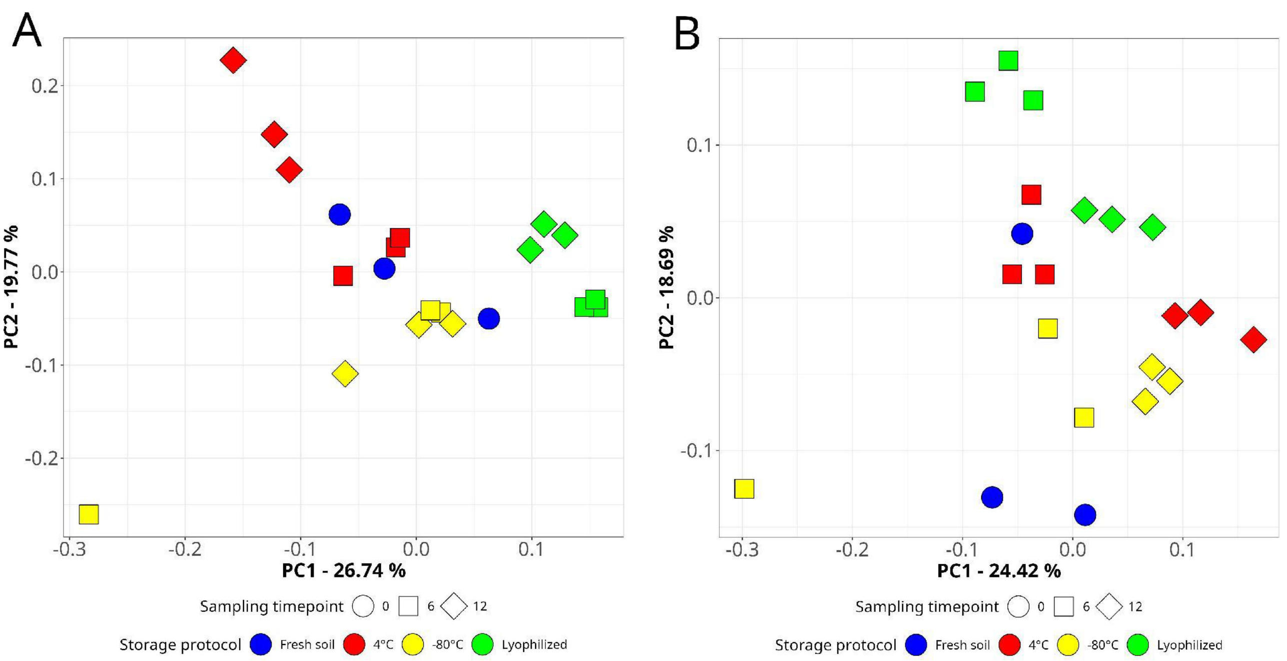

Figure S2. PCoA plot of fungal communities associated with (solarized) (A) and (control) (B) strawberry soil. Individual points represent samples, which are associated with a storage protocol (color) and sampling timepoints (shape). PC1: principal coordinate 1; PC2: principal coordinate 2. Percentage values for each axis indicate the total variance explained by each principal coordinate.

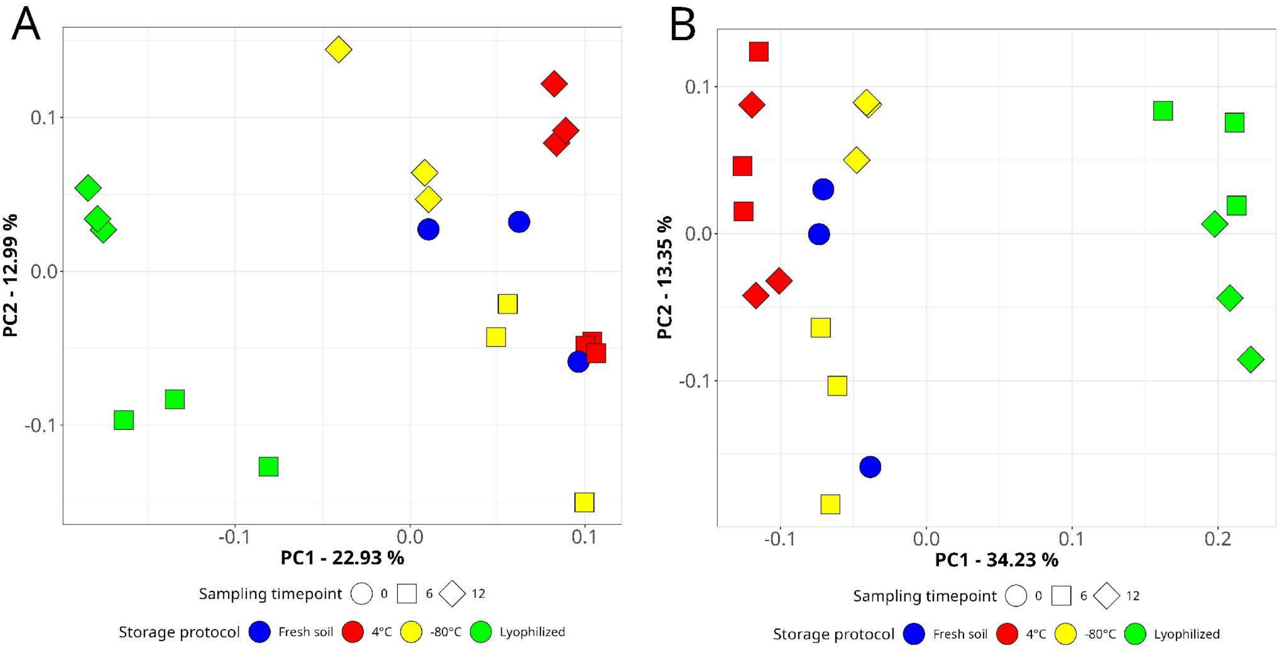

Figure S3. PCoA plot of bacterial communities associated with (solarized) (A) and (control) (B) strawberry soil. Individual points represent samples, which are associated with a storage protocol (color) and sampling timepoints (shape). PC1: principal coordinate 1; PC2: principal coordinate 2. Percentage values for each axis indicate the total variance explained by each principal coordinate.
